# Alveolar Dynamics and Beyond – The Importance of Surfactant Protein C and Cholesterol in Lung Homeostasis and Fibrosis

**DOI:** 10.3389/fphys.2020.00386

**Published:** 2020-05-05

**Authors:** Kirsten Sehlmeyer, Jannik Ruwisch, Nuria Roldan, Elena Lopez-Rodriguez

**Affiliations:** ^1^Institute of Functional and Applied Anatomy, Hannover Medical School, Hanover, Germany; ^2^Biomedical Research in Endstage and Obstructive Lung Disease Hannover, Member of the German Centre for Lung Research, Hanover, Germany; ^3^Alveolix AG and ARTORG Center, University of Bern, Bern, Switzerland; ^4^Institute of Functional Anatomy, Charité – Universitätsmedizin Berlin, Berlin, Germany

**Keywords:** surfactant protein C, pulmonary fibrosis, alveolar dynamics, lipid metabolism, alveolar macrophages, cholesterol, metaflammation

## Abstract

Surfactant protein C (SP-C) is an important player in enhancing the interfacial adsorption of lung surfactant lipid films to the alveolar air-liquid interface. Doing so, surface tension drops down enough to stabilize alveoli and the lung, reducing the work of breathing. In addition, it has been shown that SP-C counteracts the deleterious effect of high amounts of cholesterol in the surfactant lipid films. On its side, cholesterol is a well-known modulator of the biophysical properties of biological membranes and it has been proven that it activates the inflammasome pathways in the lung. Even though the molecular mechanism is not known, there are evidences suggesting that these two molecules may interplay with each other in order to keep the proper function of the lung. This review focuses in the role of SP-C and cholesterol in the development of lung fibrosis and the potential pathways in which impairment of both molecules leads to aberrant lung repair, and therefore impaired alveolar dynamics. From molecular to cellular mechanisms to evidences in animal models and human diseases. The evidences revised here highlight a potential SP-C/cholesterol axis as target for the treatment of lung fibrosis.

## Why Is Cholesterol Present in the Lung?

As recently reviewed by Zuniga-Hertz and Patel (2019), 2.32 billion years ago, the atmospheric oxygen raised (Bekker et al., 2004) leading to many changes in life. One of these changes may have been the appearance of sterols, around 2.7 billion years ago (Brocks et al., 1999; French et al., 2015) after the GOE (Great Oxygen Event) as suggested by fossil evidences. However, which is the connection between oxygen and sterols? It has been proposed that sterols emerged as an evolutionary strategy to reduce oxygen diffusion through cellular membranes, probably in a world where organisms were not ready to respond to oxidative stress yet. Interestingly, sterol biosynthesis is highly dependent on oxygen (DeBose-Boyd, 2008).

How can sterols, such as cholesterol, regulate oxygen diffusion through biological membranes? The molecular mechanism by which cholesterol can influence small solute permeation and diffusion through lipid membranes is not well understood. However, it has been proposed that the interactions between cholesterol and the acyl carbon chains in phospholipids (stabilized by Van der Waals’ forces) (Wennberg et al., 2012) create tightly packed arrangements that limits small molecule diffusion (Zocher et al., 2013). By decreasing trans-gauche rotation of the phospholipid acyl chains, membrane rigidity increases (Cassera et al., 2002; Molugu and Brown, 2019) leading to less free volume and free pockets which may accommodate oxygen, therefore reducing its flux (its partition and diffusion) through membranes (Zuniga-Hertz and Patel, 2019).

Mammals developed sophisticated organs to optimize the uptake of oxygen during the life essential breathing cycle. From conducting airways to the second biggest surface exposed to the environment, the alveolar human surface (Ochs et al., 2004). In addition, the first membrane that oxygen encounters in the mammalian lungs is a complex mixture of lipids (mainly phosphatidylcholines (PC), such as dipalmitoylphosphatidylcholine (DPPC), up to a 90%) and proteins (10%) with a 5–10% of cholesterol (14–20% mol) (Zuo et al., 2008; Bernhard, 2016), called lung surfactant (surface active agent). Interestingly two surfaces in the human body present with abnormally higher cholesterol molar ratio, and both of them are in contact with the environment tightly regulating the uptake of oxygen. On the one hand, as previously explained, lung surfactant presents around a 14–20% mol cholesterol in its composition. On the other hand the eye lens surface contains up to 35% mol of cholesterol (Raguz et al., 2008; Mainali et al., 2013), compared to a normal cell plasma membrane with a 0.5 phospholipid to cholesterol molar ratio (van Meer et al., 2008; Widomska et al., 2017). Although the main function of lung surfactant has not been related to oxygen flux control, it has been described to accelerate oxygen diffusion through a water layer (Olmeda et al., 2010), reflecting the importance of lung surfactant as potential oxygen flux regulator. The main function of lung surfactant is to reduce surface tension in order to prevent alveolar collapse during expiration and therefore stabilizing open alveoli allowing oxygen to diffuse through the lung tissue to the blood (Knudsen et al., 2017; Bates and Smith, 2018; Knudsen and Ochs, 2018). Alveolar parenchyma is thin enough to allow oxygen diffusion and is composed of a minimum of three components: (1) alveolar epithelium, where alveolar epithelial type I cells (AE1C) cover 60% of the surface with a small cytoplasm but in a long and thin disposition, whereas alveolar type II cells (AE2C) are in charge of synthesizing, secreting and regulating surfactant composition; (2) both cells sit on a very thin basal membrane; (3) to which a thin endothelium is attached on the opposite side, creating a thin but extensive air-blood barrier. The last step oxygen encounters in its way to the rest of the organs is the erythrocyte membrane. Red blood cells present a rather high cholesterol content, which comprises a 1:1 phospholipid to cholesterol ratio (Zuniga-Hertz and Patel, 2019). In addition, it has been described that oxygen diffusion through red blood cell membranes is decreased in the presence of increased cholesterol content (Buchwald et al., 2000). And therefore, reduction of cholesterol content in those membranes (such as after the use of Simvastatin for 12 weeks) improves oxygen diffusion rate (Menchaca et al., 1998).

In the long way of oxygen through the human body this review focuses on the first barrier, lung surfactant and how its components may impact the normal function of the lung. There is a particular interest in the relevance of the role of cholesterol, and more specifically, in the long described potential relation between surfactant protein C (SP-C) and cholesterol content in lung surfactant. Here, we described the state of the art at the molecular level, contrasting with data from animal models and human patients, where lung mechanics and alveolar dynamics is affected during SP-C deficiency related disease.

## Is There a Relationship Between SP-C and Cholesterol?

### Known Functions of SP-C

SP-C is the smallest (4.2 kDa) and most hydrophobic protein in lung surfactant. It accounts for ∼1% of lung surfactant mass, becoming the most abundant protein in molar terms. SP-C appeared relatively late in evolution and its sequence has remained highly conserved among species (Potter et al., 2007). The lack of any known homologous protein and its confined expression (Korfhagen et al., 1990), makes it a specific marker associated with the differentiation of lung tissue, and particularly of AE2C. SP-C exists as a 35 amino acid transmembrane protein expressed as a larger precursor (21 kDa) in AE2C cells. Structurally, it adopts a metastable α-helical structure, although its high proportion of branched residues makes it prone to adopt β-sheet structures and fibrillogenic amyloid-like aggregates (Johansson, 2001; Johansson et al., 2004; Chou and Fasman, 2006), a common feature in several interstitial lung diseases such as pulmonary fibrosis. SP-C greatly alters lipid packing in membranes influencing lipid motion and lateral distribution (Morrow et al., 1993; Dico et al., 1997). As described before, this may also influence the availability of free volume and free pockets, which may accommodate oxygen. However, a direct study of SP-C content and oxygen diffusion through surfactant membranes has not been assessed so far. SP-C also increases membrane permeability (Plasencia et al., 2004; Parra et al., 2011, 2013) and promotes interfacial lipid adsorption and lipid transfer among different lipid structures (Creuwels et al., 1993; Possmayer et al., 2001; Wang et al., 2005). SP-C is responsible for the reversible formation of multilayered stacks connected to the interfacial monolayer (Amrein et al., 1997; Wang et al., 2005). Doing so, it allows the capture of surfactant material presumably squeezed out during exhalation (area compression) and surfactant re-spreading upon inhalation (area expansion). In this process, protein palmitoylation seems to be relevant to sustain protein association to the highly compressed interfacial films reached during exhalation (Plasencia et al., 2001; Lukovic et al., 2012).

SP-C-associated functions overlap in many cases with surfactant protein B (SP-B) activity, at least when assayed in different *in vitro* models. This includes facilitating lipid adsorption (Creuwels et al., 1993; Possmayer et al., 2001; Wang et al., 2005) into the air-liquid interface or generating 3D structures that serve as a surfactant reservoir (Amrein et al., 1997; Wang et al., 2005), which store newly secreted surfactant complexes and surfactant molecules squeezed out from the interface upon compression. In this context, SP-C could be figured as a supporting molecule for SP-B function rather than an element competent by itself to assist specific features of the complex and dynamic surfactant functionality. Nevertheless, considering the extensive processing of SP-C to its mature form, its extremely conserved sequence and tissue-specific localization, and the difficulties that a cell must overcome to produce and store such a hydrophobic molecule, it is unlikely that this peptide appeared evolutionarily just as an alternative strategy to assist SP-B activities.

### SP-C and Cholesterol Relationships in the Lung Surfactant Context

Surfactant cholesterol represents a paradox regarding its origin (Orgeig and Daniels, 2001; Lopez-Rodriguez et al., 2017). Some works have suggested that it is supplied by the low and high-density lipoproteins present in blood circulation (Olmeda et al., 2017). However, other studies have failed to prove that circulating cholesterol ends up forming part of surfactant complexes (Orgeig and Daniels, 2001; Milos et al., 2016), suggesting that other possible sources must be taken into account. It is remarkable that cholesterol levels in surfactant are tightly regulated to ensure a proper breathing function, and they are able to increase and decrease extremely fast in response to changes in temperature or breathing rate (Doyle et al., 1994; Orgeig et al., 2011). This seems to imply that a cholesterol reservoir might exist in order to provide cholesterol at fast rates when an increase is required. A specific cell type, the lipofibroblast, has been suggested as a reservoir of cholesterol (Besnard et al., 2009; Torday and Rehan, 2011), although further validation is required to confirm its presence through different organisms and whether it constitutes a surfactant cholesterol storage. AE2C cells are able of producing cholesterol in peroxisomes (Batenburg and Haagsman, 1998), but alveolar macrophages (AMs) also exhibit enzymes involved in cholesterol synthesis (Baker et al., 2010b). Elucidating how cholesterol levels are regulated in the context of surfactant physiology is key to understand responses associated with several respiratory pathologies, especially those characterized by the incorporation in surfactant of abnormal cholesterol amounts such as the acute respiratory distress syndrome (ARDS) (Vockeroth et al., 2009) or pulmonary alveolar proteinosis (PAP).

The presence of cholesterol induces a marked segregation of fluid phases in surfactant (Bernardino de la Serna et al., 2004; Keating et al., 2007), and variations in cholesterol levels are known to adapt surfactant structures extremely fast to defined physiological situations (Doyle et al., 1994; Orgeig and Daniels, 2001). This evidence highlights cholesterol as a structural modulator of surfactant membranes and films. Besides, mechanisms involved in cholesterol sensing and mobilization may be evolutionary conserved to regulate cholesterol levels in surfactant. On the other hand, some studies suggest that SP-C might be involved in cholesterol regulation (Gómez-Gil et al., 2009a, b; Baumgart et al., 2010; Roldan et al., 2016, 2017), which could also be linked to the role of SP-C in lung homeostasis. Therefore, the role of SP-C in cholesterol mobilization and dynamics could be tracked back to a combined effect of protein- and cholesterol-induced alterations on membrane structure. In fact, an increase in cholesterol motion was described upon incorporation of SP-C into lung surfactant-derived vesicles (Roldan et al., 2016), an effect potentially associated to SP-C-promoted membrane-fragmenting effect (Parra et al., 2011, 2013; Roldan et al., 2016). Besides, SP-C and cholesterol have been also related to modulating membrane architecture responding in a coordinate manner to temperature changes (Roldan et al., 2017), suggesting that SP-C is involved in cholesterol mobilization by altering membrane structure (Roldan et al., 2016). In addition, taking into account that SP-C supplementation restores the functionality of cholesterol-containing films in a dynamic context (Gómez-Gil et al., 2009b; Baumgart et al., 2010), we could hypothesize that SP-C could be related to the compositional refinement of lung surfactant films from less surface-active molecules, involving cholesterol and other unsaturated phospholipids. Interestingly, observations made by Leonenko et al. (2007) could support this hypothesis. The incorporation of increasing amounts of cholesterol in a surfactant clinical preparation resulted in structurally and functionally different interfacial films (Gunasekara et al., 2005; Leonenko et al., 2007). Compression-expansion isotherms allowed the determination of lipid loss from the interfacial film upon compression, which were considerably low for physiological cholesterol amounts, permitting the re-establishment of a functional interfacial film (Gunasekara et al., 2005; Leonenko et al., 2007). Shifting that to SP-C function, lipid loss occurring for physiological cholesterol levels could be associated with a SP-C-dependent cholesterol refinement, in which SP-C-induced lipid reorganizations could be involved. However, lipid loss increased substantially for cholesterol concentrations beyond physiological levels, showing a markedly different film structure and impaired functionality (Gunasekara et al., 2005; Leonenko et al., 2007). For these supra-physiological cholesterol levels, the SP-C amount present in a clinical surfactant preparation like the one tested in this study (∼1 wt%), could be likely insufficient to overcome cholesterol impairing effects and the irreversible film collapse. Supplementation of these films with different SP-C amounts would provide valuable information to confirm SP-C effects on cholesterol mobilization.

SP-C/cholesterol relationships might be involved in surfactant refinement with a homeostatic purpose in the lung. Both, cholesterol-loaded vesicles, as well as their combination with SP-C, led to a higher lipid engulfment by AMs (Ruwisch et al., 2020). Increased cholesterol content, however, seemed to increase lipid uptake by AMs regardless of the presence of SP-C. Vesicle size and membrane fluidity can affect lipid uptake by AMs (Justice et al., 2014), and thus, the combined effect of cholesterol and SP-C on membrane structure and fluidity (Roldan et al., 2016, 2017) is a factor that must be taken into account. The study of the transcriptional response of MH-S cells to lipid administration revealed that genes associated to lipid metabolism and cholesterol transport were altered upon lipid uptake. Changes in gene expression appear to depend on lipid composition, including the presence or absence of SP-C and/or cholesterol. However, specific effects of cholesterol and SP-C require a thorough and extensive analysis, which also warrant a deeper exploration of the poorly studied pathways for cholesterol synthesis, degradation and mobilization in AMs.

SP-C could then be considered as a pivotal molecule linking cholesterol and lipid homeostasis, lung immunity, lung surfactant biophysical activity and potentially oxygen diffusion regulation, which would explain its particular features, lung tissue specific localization and sequence conservation along evolution. Several questions remain to be answered, including how SP-C effects are coordinated with SP-B function to modulate surfactant activity; which are the molecular mechanisms and SP-C structural determinants involved in membrane reorganizations and how cholesterol levels in surfactant are sensed and regulated regarding SP-C function by both AE2C and AMs. Future research assessing these questions would constitute an essential piece to understand the molecular mechanisms ruling lung surfactant behavior and its implication in respiratory pathologies, generating valuable information useful to improve the current clinical preparations applied for surfactant replacement therapy.

## SP-C, Cholesterol and Alveolar Micromechanics

### SP-C Deficiency Predisposes to Alveolar Instability

**FIGURE 1:**
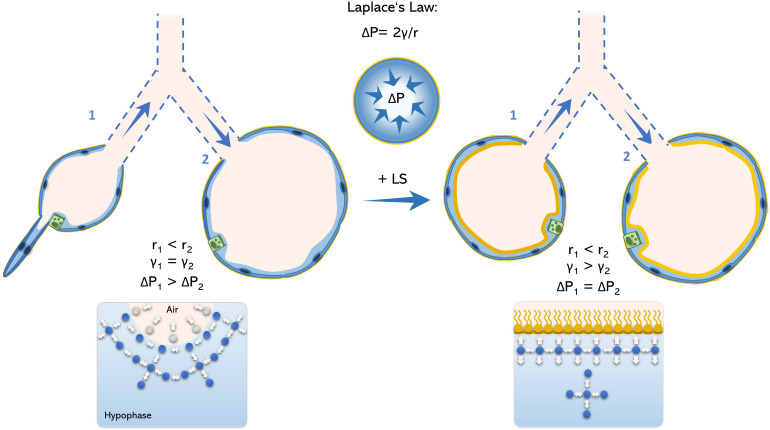
Schematic model of Young-La Place law in the acinar airways. The inner pressure of an idealized spherical alveolus is defined by the Young-La Place law as ΔP = 2γ/r. Therefore, alveoli with small radius (alveolus 1) comprise higher internal pressures in relation to alveoli with larger radius (alveolus 2). Accordingly, alveolus 1 would tend to collapse toward alveolus 2 under the absence of a functional surfactant film, what permits alveolar size heterogeneity **(left panel)**. However, in the presence of surfactant, surface tension is reduced as intermolecular forces are homogenously generated between water-water- and water-air phase, avoiding bending of the air-liquid interface and avoiding alveolar collapse **(right panel)**. LS, lung surfactant.

According to LaPlace law, the intra-alveolar pressure of and idealized isotropic alveolus is inversely proportional to its radius. Therefore, at end-expiration, the breathing stage in which the intra-alveolar volume reaches its minimum (ignoring rare occurring phenomena like Pendelluft; Tabuchi et al., 2016), the intra-alveolar pressure reaches its maximum. Under physiologic conditions, end-expiratory pressure is homogenously distributed among the alveoli and surface tension is low enough preventing alveolar collapse. To maintain alveolar pressure throughout the lung constant, surface tension also changes accordingly to alveolar radius (Figure 1).

However, during expiration the surfactant film collapses and thus, end-expiratory surface tension may drastically increase and lead to higher intra-alveolar pressures as well as a rather heterogeneous inter-alveolar pressure distribution. As the intra-alveolar pressure is *per se* higher in small-radius alveoli (relative to its larger neighbors) by its anatomy, this may increase the likelihood of alveolar collapse. Hence, it has been described that the result of surfactant dysfunction in a murine model of AE2C apoptosis is ductal airspace over distension together with alveolar collapse (Mouded et al., 2009). On the one hand, this may explain the increased vulnerability of small subpleural alveoli to surfactant dysfunction. On the other hand, points toward an essential role of alveolar collapse in fibrotic remodeling, as collapsing alveoli was proposed to be the initiating hotspot in pulmonary fibrogenesis (Mai et al., 2016; Knudsen et al., 2017; Petroulia et al., 2018).

### Alveolar Collapse – The Springboard for Lung Mechanic Impairment

The central role of alveolar collapse in the development of diseases such as lung fibrosis is not a novel finding and was already stated by Myers and Katzenstein (1988). Moreover, alveolar collapse induced by surfactant dysfunction has also been well described in the field of ARDS and acute lung injury (ALI) (Bates and Smith, 2018; Nieman G.F. et al., 2018). Although the increase in surface tension and alveolar instability is much more pronounced in ARDS (Smith et al., 2017; Autilio and Pérez-Gil, 2018) than under SP-C deficiency, leading to a rather acute than chronic respiratory pathology, ARDS as well as rather chronic lung fibrosis may be associated with a similar underlying disease mechanism. This is highlighted by the fact that alveolar collapse, respiratory distress and concomitant ventilation in-homogeneities haven been demonstrated to be present in patients from both diseases (Todd et al., 2015; Al-Saiedy et al., 2018; Autilio and Pérez-Gil, 2018; Petroulia et al., 2018). Moreover, pulmonary fibrosis is also seen in later disease stage of ARDS patients, resembling an aberrant pulmonary repair mechanism in response to lung injury (Hasan et al., 2017), which has also been stated to be the major mechanism of disease in idiopathic pulmonary fibrosis (IPF) patients (Snijder et al., 2019).

**FIGURE 2:**
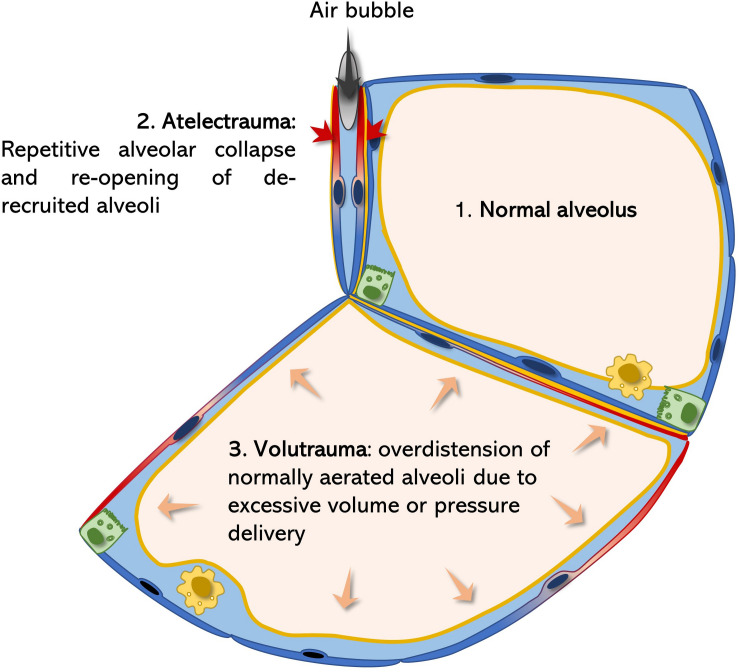
Surfactant dysfunction related atelectrauma and volutrauma. According to the La Place law (Figure 1) surfactant dysfunction predisposes alveoli for collapse. Alveolar micro-atelectasis causes septal distortion of neighboring alveoli due septal, interalveolar architectural dependence. Thereby the collapsed alveolus acts as a stress concentrator leading to injurious mechanical deformation of neighboring epithelial cells (blue-red color transition) during the breathing cycle (volutrauma). Meanwhile, every time a bubble of air is forced into the atelectatic, lining-fluid filled alveolus during inspiration, as a result of increasing transpulmonary pressures, extremely damaging shear stresses are generated on the epithelium (atelectrauma), further potentiating damage of the alveolar epithelium (Bates and Smith, 2018; Rühl et al., 2019).

Atelectrauma and volutrauma are two mechanism contributing to tissue injury in ARDS (Figure 2). In the injured lung, repetitive alveolar collapse (atelectrauma) is caused by increased surface tension (induced by various agents). During the inspiration phase, the transpulmonary pressure gradient increases until the alveolar pressure of the closed (de-recruited) alveolus is exceeded and is re-opened (recruited). If the maximal generated transpulmonary pressure fails to exceed the intra-alveolar pressure, the alveolus remains collapsed and its alveolar walls stay adjacent to each other. This model is supported by findings of Bachofen and Schürch, who described alveolar wall stretching and ductal airspace over distension on electron microscopic images resulting from surfactant inactivation-induced alveolar collapse (Bachofen and Schürch, 2001). Considering the mechanism of atelectrauma, the alveolar lining fluid should not be neglected. Weibel and Gil first described the alveolar lining lavage as an interfacial film and sub-interfacial liquid reservoir defined as the hypophase (Weibel and Gil, 1968). As the alveolus deflates during expiration, its defining septa start to fold. The folding septal branches are continuously filled with this alveolar lining fluid during expiration (Rühl et al., 2019). Under increased surface tensions the alveolus is further de-recruited leading to more pronounced septal folding and enhanced inter-septal liquid accumulation. During inspiration, an increasing transpulmonary pressure is generated by the respiratory muscles (Mead et al., 1970; Gattinoni et al., 2017). As soon as this pressure exceeds the end-expiratory intra-alveolar pressure, a bubble of air is forced through the lining fluid between the septal folds in order increase alveolar volume by forcing septal de-folding. During this process mechanical axial shear stresses are generated with the air-bubble moving toward the lining fluid, leading to injurious deformation of AEC2 (Bilek et al., 2003). Of note, the cell damaging force vectors strongly increase with rising surface tension at the interface of the lining fluid, as the speed of the bubble is highly reduced (Cassidy et al., 1999; Ghadiali and Gaver, 2008). Meanwhile, AEC2 injury impairs the epithelial functional integrity resulting in accumulation of alveolar edema and reduced rates of surfactant production. Thus, starting a vicious cycle leading to continuously increasing surface tension and epithelial injury, resulting in AEC2 hyperplasia and fibrosis (Fehrenbach, 2001; Mouded et al., 2009; Sisson et al., 2010) (Figure 2).

In the healthy parenchymal lung architecture, tissue stress and stretch occurring during breathing are homogenously distributed. However, if certain alveolar regions become atelectatic, homogenous ventilation is significantly impaired. Alveolar collapse leads to microscale air redistribution, resulting in an inhomogeneous alveolar size distribution before inspiration (Mead et al., 1970). According to alveolar anatomy, single septal walls usually build the parenchymal border of two neighboring alveoli, also known as alveolar interdependence (Knudsen and Ochs, 2018). If one of these alveoli collapses, septal distortion of the adjacent alveoli leads to rising mechanical stresses (Mead et al., 1970). Hence, atelectatic alveoli act as stress concentrators and amplify the applied stresses and strains of their neighbors (Bates and Smith, 2018). During inspiration and increasing lung volumes, this phenomenon becomes even more relevant: transpulmonary pressures generated during inspiration will initially rather lead to over inflation of the already enlarged, distorted alveoli than reopening the atelectatic ones. This causes further alveolar over distension during inspiration. During over distension, an alveolus is first fully de-folded before its wall is stretched. Therefore, the effective septal deformation (= strain) strongly depends on the inspired/applied tidal volume and the alveolar “baseline” shape during resting expiratory position. Usually mechanical stretch primarily occurs under rather high pressures reaching over the upper inflection point of the pulmonary pressure-volume curve (Knudsen and Ochs, 2018). However, when inflating a healthy lung beyond total lung capacity (TLC) of 80% the extracellular matrix components of the alveolar septa, collagen and elastin, become the major stress bearing components, thus mechanical forces start acting on the septal interstitium (Bachofen et al., 1987; Knudsen and Ochs, 2018). Moreover, single vulnerable alveoli in proximity to stress concentrators, may already get stretched at physiologic low tidal volumes (Mead et al., 1970). In line with this, Kollisch-Singule et al. (2015) found pulmonary injury in surfactant depleted pig lungs already at physiologic tidal volumes (6 ml/kg bodyweight), while pigs with maintained surfactant function, required a drastically higher degree of volutrauma to induce relevant lung injury (30 ml/kg bodyweight). In the ARDS lung atelectrauma and volutrauma act synergistically to induce vascular leakage. Smith et al. (2013, 2017) elegantly demonstrated that initial epithelial injury by atelectrauma predisposes septal walls for volutrauma, leading to significant leakage of the gas-blood barrier. Ventilated mice lungs only developed high concentrations of BALF protein levels, an indicator for gas-blood barrier integrity, when ventilated at PEEP (positive end-expiratory pressure) of 0cmH2O (atelectrauma) in combination with mid or high tidal volumes (volutrauma) (see Figure 2). Meanwhile, neither atelectrauma nor volutrauma alone were sufficient to induce pulmonary edema (Smith et al., 2017). As alveolar epithelial cell (AEC) damage has been demonstrated to precede edema formation (Rühl et al., 2019), the absence of alveolar edema in the SP-C deficient lung (Ruwisch et al., 2020), does not rule out the relevance of this protein in preventing alveolar collapse and alveolar over distension, which commonly lead to microscale tissue injuries. Interestingly, hyperplastic AEC2 are present in a global SP-C knock out (KO) mouse model, which may resemble a reaction to chronic mechanical stress (Glasser et al., 2003; Ruwisch et al., 2020). Moreover, surfactant dysfunction in a chronic murine model of AEC injury impressively demonstrated the long-term effects of chronic airway instability to cause microarchitectural air re-distribution resulting in an emphysema-like phenotype, which is characterized by collapsed alveoli and enlarged alveolar ducts (Mouded et al., 2009). Furthermore, repetitive insults during dynamic strain have been related to more harmful properties on AEC integrity than constant high static strains (Nieman G. et al., 2018). This may further emphasize the relevance of airway instabilities, in the absence of volutrauma, in a rather chronic setting, as occurs in IPF patients, where recruitment/de-recruitment cycles take place over a long period of time. In line with this, “velcro-crackles” have been often described to precede computer tomographic changes in IPF lungs. Indeed, they are considered the auditory correlate of damaging energy-rich alveolar re-openings during inspiration, supporting the clinical relevance of impaired alveolar micromechanics in fibrosing lung diseases (Vyshedskiy et al., 2009).

At the lung mechanical macroscale level, alveolar collapse and surfactant dysfunction exhibit notable influence on the lung’s viscoelastic properties. Collapsed airways require high amounts of energy to be reopened, while dysfunctional surfactant increases the hysteresis effect in inflating lungs by rising surface tension. This effect results in parenchymal stiffening, increasing levels of pulmonary tissue elastance (Smith et al., 2013; Birkelbach et al., 2015). During inspiration, the energy applied is rather dissipated creating injurious axial force vectors on the airway epithelium than being stored in the septal elastic fiber network. Therefore, pulmonary tissue damping rises accordingly. In line with this, data from our group demonstrated a significant increase in tissue damping and tissue elastance in 10 weeks old SP-C deficient mice in contrast to age-matched control mice, supporting the concept of alveolar collapse as an initial trigger (Ruwisch et al., 2020). During aging, redistribution of air as well as ECM (Extracellular Matrix) remodeling may alter lung mechanics consistent with findings of Glasser et al. (2001, 2003). Indeed, this work emphasized aberrant tissue hysteresivity (the quotient of energy dissipative forces and lung elastance) at low PEEP, a condition where alveoli are prone to collapse. At those low lung volumes, surfactant becomes the major defining factor of pulmonary breathing mechanics (Bachofen et al., 1987). Moreover, pressure-volume loops demonstrated an increased pulmonary hysteresis, which also reflects increased energy dissipation during inflation of the lung. Gattinoni et al. (2017) already suggested this energy to be potentially harmful for the pulmonary tissue, as it is not reused for elastic recoil properties. Persistent septal micro injury may be, in turn, a relevant factor for altered AEC2 biology, aberrant wound repair, pulmonary inflammation and interstitial fibrotic remodeling. Phenomena described in a number of children suffering interstitial lung disease (chILD), who showed reduced or absent levels of mature SP-C (Cong et al., 2017), as described in the next sections.

### Establishing Injurious Mechanical Stress as a Driving Factor for Fibrogenesis

Under healthy conditions and homogenously distributed lung stress, plasma membrane injury of AEC is normally seen. Indeed, AEC are highly capable of Ca^2+^ and lysosome dependent repair mechanisms as well as membrane folds for preventing cellular damage (Cong et al., 2017). However, lysosomal stress as seen in Hermansky-Pudlack Syndrome (HPS) patients may impair cellular repair, favoring profibrotic wound repair and the development of unusual interstitial pneumonia (UIP) (Ozyilmaz et al., 2013; Kook et al., 2016). Moreover, if high parenchymal stress leads to plasma membrane defects of more than 1 μm, aberrant AEC wound repair cascades are induced (Cong et al., 2017). Very high strains induced by pressure above 40cmH_2_O have been shown to be sufficient to cause membrane blebbing and cellular apoptosis (Dreyfuss et al., 1999).

Typically, AEC injury response includes signaling of several mechano-transduction pathways including the transforming growth factor β1 (TGF-β1), Wnt β-catenin, sonic hedgehog (Shh), and the Notch-midkine signaling pathways as well as induction of endoplasmic reticulum (ER) stress. Interestingly, all these pathways have been interlinked with the induction of profibrotic genes, epithelial-to-mesenchymal transition (EMT) or increased ECM deposition. Moreover, ER stress is known to compromise the secretory capacity of AEC2, impairing surfactant metabolism, which initiates a vicious cycle of alveolar instability and stress-mediated AEC injury (Cong et al., 2017). In a murine model of chloride acid (HCl) induced surfactant depletion, impaired lung mechanics was characterized by increased tissue elastance. This was followed by an increased expression of various mesenchymal markers, such as α-smooth muscle actin (α-SMA) and vimentin in AEC2, whereas the expression of epithelial cell markers, including pro-SP-B, were reduced (Cabrera-Benítez et al., 2012; Mao et al., 2017). Consequently, EMT may be a potent second hit even further compromising: (1) surfactant function and; (2) the regenerative capacitiy of the pulmonary epithelium further directing the damage response toward fibrosis. TGF-β1 is expressed by both AECs as well as resident inflammatory cells like AM (Saito et al., 2018). In addition, a large pool of latent-inactive TGF-β1 is located as a complex with latency associated peptide 1 (LAP-1) and latency TGF-β1 binding protein-1 (LTP-1) in the ECM (Hinz and Suki, 2016; Saito et al., 2018). In the healthy lung, during inspiration, ECM fiber stretching is not sufficient to release active TGF-β1 from its inhibitory binding complex. However, as soon as pulmonary tissue stiffening occurs, interstitial fibroblasts are primed toward myofibroblast differentiation by expressing contractile actin-myosin elements (Zhou et al., 2013). This drastically increases the likelihood of TGF-β1 release upon parenchymal strain, as myofibroblast are able to bind LAP-1 via αV-integrins (Hinz, 2012; Hinz and Suki, 2016). Interestingly, increased epithelial injury resulted in increased expression of epithelial αVß6-integrins, further potentiating the release of TGF-β1 by mechano-transduction (Sheppard, 2015). In line with this, Froese et al. (2016) showed increased expression and release of TGF- β1 in fibrotic IPF lungs, which are characterized by parenchymal stiffening. Importantly the pressures generated by Froese and his team rather reflected gradients comparable to pressure induced by spontaneous breathing than high unphysiological gradients, emphasizing the role of spontaneous breathing as a relevant source of mechanical stress-released active TGF-β1 (Hinz and Suki, 2016). Nevertheless, the role of non-fibrotic but rather collapse-induced parenchymal stiffening in the context of extracellular (EC) TGF-β1 release to date has not been clarified. Finally, TGF-β1 overexpressing mice have been shown to develop surfactant dysfunction and high surface tension (Lopez-Rodriguez et al., 2016) including down-regulation of SP-C expression, by interfering with its transcription factor activity. In this context, considering the afore mentioned section, it becomes clear that an EC TGF-β1 pool bears the potential of an additional devastating loop by further compromising lung mechanics, potentiating epithelial injury.

### Inflammatory Response Under Impaired Lung Mechanics – Where Sterols Come Into Play

The role of inflammation and inflammatory cells in fibrogenesis has been controversial over the last decades. Although anti-inflammatory medications have produced devastating clinical results in a IPF clinical trials (Raghu et al., 2006; Idiopathic Pulmonary Fibrosis Clinical Research Network et al., 2012), the role of immune cells, like AMs, in lung fibrogenesis and pulmonary tissue repair has been repetitively underscored (Wynn and Barron, 2010; Wynn et al., 2013; Romero et al., 2014; Jin et al., 2018; Smigiel and Parks, 2018; Puttur et al., 2019). A promising link between mechanical stretch and inflammatory response may be the release of adenosine triphosphate (ATP) (Cong et al., 2017; Hasan et al., 2017). Physiologically, mechanical stretch of AEC1 transmits the release of ATP via P2XR7, functioning as a stretch sensor (Patel et al., 2005; Mishra et al., 2011). However, under prolonged alveolar injury concentrations of ATP and its related nucleotides sharply rise, finally overwhelming the degradative enzymatic machinery. Hence, levels of ATP continuously stay high in the early exudative stage of ARDS until the expression of its degrading ecto-enzyme catches up (Hasan et al., 2017). Under these circumstances, ATP and its metabolites act as damage associated molecular patterns (DAMPs). This results in pro-inflammatory signaling on the one hand, but interestingly also in the induction of various profibrotic pathways on the other hand. In fact, high levels of EC ATP enhanced TGF-β1 expression and deposition of collagen 1 and fibronectin in fibroblasts via P2XR7 signaling *in vitro* (Wang et al., 2003; Qu et al., 2009). Meanwhile, ATP signaling via the same receptor enhanced the release of profibrotic tissue-metalloprotease inhibitor 1 (TIMP1), which attenuates the function of ECM-degrading enzymes, resulting in increased interstitial amounts of ECM, a hallmark of lung fibrosis (Gu and Wiley, 2006). Interestingly, elevated levels of TIMP1 have also been observed in bronchoalveolar lavage fluid (BALF) of IPF patients, whereas expression of Timp1 was induced in 10 weeks old SP-C deficient mice (Beeh et al., 2003). In addition, both Ca^2+^ and ATP are well-known stimulating agents of surfactant secretion (Voyno-Yasenetskaya et al., 1991; Dico et al., 1997; Patel et al., 2005; Dietl et al., 2012). This may reflect a counteracting response, where surfactant secretion may be enhanced during high release of ATP and Ca^2+^, during injury.

**FIGURE 3:**
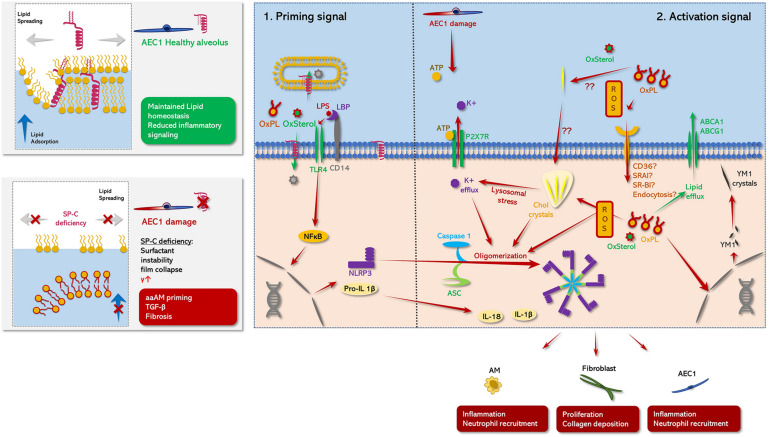
Schematic role of SP-C in pulmonary metaflammation. **(Left panel**) comparison between the status of AE1C in the presence (healthy alveolus, top panel) or absence (damaged alveolus, bottom panel) of SP-C. **(Right panel**: **1) Priming signal:** TLR4 signaling is abrogated under the presence of mature SP-C. On the one hand, SP-C interferes with the complex formation of LBP and CD14 potentially attenuating TLR4 mediated NLPR3 priming in response DAMP/PAMP stimuli. This ant-inflammatory effect may be further potentiated by the N-terminal segment of SP-C transferring TLR4 activators either into neighboring phospholipid microsomes or directly to the cytoplasmic compartment of the macrophage (Chaby et al., 2005; Garcia-Verdugo et al., 2009). Moreover, maintained surfactant homoeostasis/surfactant catabolism counteracts the accumulation/formation of oxidized lipid species and cholesterol clefts on the one hand, and minimizes atelec- and volutrauma on the other hand (Ruwisch et al., 2020), likely diminishing the release of DAMPS like ATP on the other hand. Together this prevents the buildup of inflammasome oligomerizing stimuli, what may at least partly explain the aberrant inflammatory response of SP-C KO mice to various inflammatory stimuli in contrast to WT mice (Glasser et al., 2008, 2013a). **(Right panel: 2) Activating signal:** NLRP3 inflammasome comprises an initial NFκβ related priming phase, preceding a secondary activation signal (Swanson et al., 2019): In the first step various proinflammatory DAMPS (oxysterols, oxidized PL species) or PAMPS (LPS) activate macrophages via TLR-4 signaling via an either LPS binding protein (LBP)/CD14 dependent or independent way (Chaby et al., 2005; Garcia-Verdugo et al., 2009). Thereby, LBP facilitates the binding of e.g., LPS to CD14, what accelerates LPS related TLR4 signaling. TLR4 signaling in turn drives NFκB-mediated expression of NLRP3, pro-IL-β1 and proIL-18 (Priming phase) (Gasse et al., 2007; Swanson et al., 2019). In the second step, multiple potential hits including PAMPS like extracellular ATP (Mariathasan et al., 2006; Swanson et al., 2019), intracellular cholesterol clefts (Ertunc and Hotamisligil, 2016), oxysterols and oxidized phospholipid derived mitochondrial oxidative stress (Fessler, 2017; Manon et al., 2018) induce previously synthetized NLRP3 to form oligomers with caspase 1 and ASC leading to mature inflammasome formation. Thereby, increased level of extracellular ATP may be derived from mechanically stressed AEC1 (Hasan et al., 2017), leading to an inflammasome activating K^+^ efflux via P2X7R. Meanwhile, SP-C deficiency related dysfunctional surfactant catabolism and surfactant dysfunction may favor the generation of oxidized PL species and the formation of cholesterol clefts (Fessler and Summer, 2016; Ruwisch et al., 2020). Likewise, these cholesterol crystals cause lysosomal stress, which in turn resembles another potent driving factor of inflammasome formation via induction of a K^+^ efflux. Finally, the active NLRP3 inflammasome converts inactive proIL-1β and proIL-18 into their active form IL-1β and IL-18. IL-1β promotes fibrotic remodeling (Gasse et al., 2007; Cassel et al., 2008; Wree et al., 2014; Lv et al., 2018). Meanwhile, another effect of accumulation of injurious lipid species inside the macrophages may also prime their hosts toward a profibrotic aaAM-phenotype (Romero et al., 2014) via induction of several aaAM related genes including, chitinase-like-3 (YM1), which has also been described to form electron dense crystals in various alternative activation of macrophages-disease models (Hoenerhoff et al., 2006; Mora et al., 2006), what may result in a profibrotic feed-forward loop (Smigiel and Parks, 2018).

Alternatively activated alveolar macrophages (aaAMs) are mainly characterized by expression of resistin-like alpha (Relm-α), chitinase-like proteins (YM1), inducible nitric oxide synthase (NOS-2) or arginase 1 (Arg-1) (Wynn and Barron, 2010). Their profibrotic potential derives primarily through expression of TGF-β1 and the activation of latent TGF-β1 via matrix-metalloproteases (e.g., MMP-9) (Xing et al., 1997; Schmid-Kotsas et al., 1999; Cameron et al., 2001; Karlmark et al., 2009; Lin et al., 2009). Moreover, aaAMs haven been shown in topographic proximity to myofibroblasts, being highly capable of collagen secretion (Ramm et al., 1998; Leicester et al., 2004; Thompson et al., 2008). Finally, aaAMs prime the alveolar micromilieu toward fibrosis by secreting various pro-fibrotic cytokines including IL-4, IL-10, IL-13 and the rather pro-inflammatory cytokine IL-1β. Of note, induced expression of YM1, a chitinase-like protein 1 (Bargagli et al., 2007; Terèelj et al., 2009; Wynn and Barron, 2010), but also in SP-C deficient mice (Glasser et al., 2003). Increased accumulation of macrophages with induced TGF-β1 expression was seen in bleomycin-challenged SP-C deficient mice in areas with significant parenchymal distortion. This further emphasizes the link between mechanical stress and macrophage regulation (Lawson et al., 2005). Interestingly, this misbalance between classical and alternative differentiation was not solely dependent on SP-C deficiency, but also specific to the genetic 129/Sv6 background. The 129/Sv6 strain is characterized by an increased dietary cholesterol accumulation and has been described to develop foamy, lipid-laden macrophages with highly induced YM1 expression with age (Jolley et al., 1999; Hoenerhoff et al., 2006). Curiously, SP-C deficient mice as well as patients suffering mutant SP-C derived interstitial lung diseases (ILDs) related to mutations of SP-C, showed similar foamy macrophages in BALF and tissue as well as abundant cholesterol clefts (Glasser et al., 2003; Hamvas et al., 2004; Abou Taam et al., 2009; Ruwisch et al., 2020). Taken together, this suggests a SP-C deficiency and cholesterol induced axis resulting in dysregulated macrophage metabolism and in misbalanced alternative differentiation (Figure 3). Cholesterol clefts are present in aging 129/Sv4 mice (Hoenerhoff et al., 2006), SP-C deficient mice (Glasser et al., 2003; Ruwisch et al., 2020) or aaAMs in human herpes virus (HHV)-induced lung fibrosis (Mora et al., 2006). Hence, this may be a potential morphological biomarker of dysregulated alternative activation and an indicator for lung fibrosis. However, the exact interplay between cholesterol and SP-C and macrophage regulation has not been yet elucidated. Nevertheless, studies from our group, suggested SP-C to be necessary for maintaining cholesterol homeostasis in an AM cell line (MHS cells) (Ruwisch et al., 2020). In line with this, Romero and colleagues recently proposed the profibrotic potential of a lipid related paracrine axis between macrophages and epithelial cells (Romero et al., 2014). Interestingly, their team demonstrated the presence of foamy, lipid-laden macrophages overexpressing YM1 and TGF-β1 in bleomycin induced lung injury, which has been already associated with reduced expression of the *Sftpc* and *Sftpb* gene (Schmidt et al., 2004; Lutz et al., 2015). Romero’s team further showed oxidized phospholipids to be another crucial inductor of foam cell formation and alternative differentiation. The role of oxidized lipid species to induce foam cell formation in macrophages has been extensively studied in the field of atherosclerosis (Ertunc and Hotamisligil, 2016). Circulatory macrophages located in atherosclerotic plaques are frequently challenged with oxidized low-density lipoprotein (LDL), oxysterols or cholesterol. These lipids are endocytosed via various receptors, including CD36 and scavenger receptors. Accumulation of toxic lipid species decompensates the metabolic capacity of those macrophages, leading to cholesterol cleft formation and induction of necrotic factor kappa beta (NFκB) related cellular stress pathways. One central element in the so called “metaflammatory” process. This is the formation of the NOD-, LRR- and pyrin domain-containing protein 3 (NLRP3) inflammasome, resulting in the production of pro-inflammatory cytokines including IL-1β and IL-18 (Duewell et al., 2010; Ramji and Davies, 2015). Importantly, macrophage derived IL-1β and inflammasome activation have been referred to as key features in various forms of lung fibrosis including IPF (Zhang et al., 1993; Kolb et al., 2001; Gasse et al., 2007; Wynn and Barron, 2010; Eldridge et al., 2017).

In addition, cholesterol-induced “metaflammation” in macrophages may also be not only important in development of atherosclerosis but seems to be also highly relevant in the lung. For example, AMs are central elements in surfactant catabolism (comprehensively reviewed in Lopez-Rodriguez et al., 2017), and are continuously challenged with various phospho- and neutral lipids. Therefore, knocking down cholesterol transporters, such as ATP binding cassette transporter A1 (ABCA1) and G1 (ABCG1) leads to impaired cellular cholesterol efflux and cholesterol accumulation. In addition, those animal models showed respiratory distress, impaired surfactant metabolism and cholesterol cleft containing, lipid-laden AMs (Thomassen et al., 2007; Baker et al., 2010a; Chai et al., 2017; Fessler, 2017). Moreover, impaired reverse cholesterol transport in apolipoprotein E (ApoE) and apolipoprotein A1 (ApoA1) deficient mice was also linked to increased pulmonary oxidative stress and inflammation (in ApoE KO mice) as well as to foam cell formation and lung fibrogenesis (in ApoA1 KO mice) (Yao et al., 2016). Interestingly, surfactant function as well as metabolic integrity of AM was restored after application of cyclodextrin, a cholesterol sequestering-drug, underlining the injurious potential of cholesterol for surfactant function and pulmonary lipid homeostasis (Gunasekara et al., 2010). The finding of cholesterol clefts and lipid laden macrophages in patients with SP-C mutations suggests impaired cholesterol homeostasis to be of relevance for development of lung disease in these patients (Hamvas et al., 2004; Abou Taam et al., 2009; Mechri et al., 2010; Cottin and Cordier, 2011; Litao et al., 2017).

Cholesterol overloaded macrophages exhibited an increased Toll-like receptor 4 (TLR-4) signaling mediated immune response as well as elevated expression levels of IL-1β, emphasizing a potential role of NLRP3 inflammasome activation in pulmonary macrophages (Fessler, 2017). The NLRP3-inflammasome consists of three elements including the NLRP3, procaspase 1 and the apoptosis speck-like protein (ASC) containing a caspase recruitment domain (CARD), which links NLRP3 with procaspase 1 (Robert et al., 2016). In order to generate full activation of the inflammasome complex usually two independent signals are needed (Figure 3): (1) a NFκβ priming signal TLR-4 signaling [enhanced by lipopolysaccharide (LPS) or oxidized phospholipid species] or the presence of cholesterol crystals (Bauernfeind et al., 2009; Ertunc and Hotamisligil, 2016; Fessler and Summer, 2016; Nakayama, 2018; Saito et al., 2019) and; (2) purinergic signaling as may result from mechanical stress induced AEC1 injury (Hasan et al., 2017). Elevated levels of ATP have been found in BALF of patients with lung fibrosis, further supporting the idea of EC ATP as a potential inflammasome co-activator (Riteau et al., 2010). Hence, the NLRP3 inflammasome may resemble a crucial link between impaired lung mechanics and aberrant lipid metabolism in fibrosing lung diseases. Interestingly, IPF patients have been described to have elevated levels of unsaturated PC, a PC variant which highly susceptible for oxidative stress (Fessler and Summer, 2016). In combination with the fact, that IPF occurs predominantly in the elderly and has been interlinked with cigarette smoking, mitochondrial stress and cellular senescence, factors driving the generation of reactive oxygen species (ROS). Thus, the IPF lung appears as a hotspot for lipid oxidation (Lederer and Martinez, 2018). Elevated levels of ROS and increased levels of cholesterol in surfactant are also present in bleomycin-induced lung fibrosis (Allawzi et al., 2019), highlighting the relevance of ROS, surfactant oxidation and foam cell formation in fibrogenesis. This is further supported by the presence foamy, Oil-Red-O positive cells in BALF of patients not only with *Sftpc* mutations, but also in patients with sporadic interstitial fibrosis (Basset-Léobon et al., 2010).

Nevertheless, SP-C deficiency may play a distinct role in foam cell formation. Not only is SP-C crucial for stabilizing cholesterol-containing surfactant films (Gómez-Gil et al., 2009a, b) and maintaining physiologic mechanical lung properties but also it seems to interfere with TLR-4 signaling. *In vitro* studies demonstrated that SP-C attenuates LPS induced cytokine production via TLR-4 signaling in a macrophage cell line, suggesting SP-C to play a role in innate immunity (Augusto et al., 2003). Thereby, SP-C seems to compromise the affinity of TLR-4 to LPS via a complex interplay with CD14 (Chaby et al., 2005). However, Garcia-Verdugo et al. (2009) also proposed a CD14 independent mechanism, by which the transfer of LPS into liposomes mediated by the N-terminal SP-C segment would prevent LPS binding to TLR4. This results in a reduced cytokine production upon LPS stimulation (Garcia-Verdugo et al., 2009). In line with these findings, SP-C KO mice exhibited an increased inflammatory response upon LPS challenge characterized by increased macrophage accumulation and higher expression levels of IL-1β, IL-6 and tumor necrosis factor alpha (TNF-α) (Glasser et al., 2013a). Interestingly, SP-C containing phospholipid vesicles reduced TLR-4 signaling in these mice. Noteworthy, this immunomodulatory role of SP-C seems to not to be restricted to LPS and bacteria-induced inflammation, as SP-C also demonstrated an anti-inflammatory role under pathogen-free conditions (Jin et al., 2018). Taken all together, this suggest that SP-C may not only orchestrate the activation of TLR-4 in an infectious setting, but it also has an impact on TLR-4 signaling in macrophages induced by oxidized surfactant lipids, a question that, to date, remains unclear.

Assuming this hypothesis (Figure 3), SP-C deficiency could predispose to TLR-4 activation by various oxidized cholesterol and PL species as well as their uptake. This results in proinflammatory signaling and NLRP3 inflammasome activation in macrophages (Ertunc and Hotamisligil, 2016). Impaired lung mechanics induced by SP-C deficiency may further promote inflammasome activation by ATP release and at the same time, lead to accelerated surfactant aggregate conversion (Veldhuizen et al., 1996, 1997; Vazquez De Anda et al., 2000; Maitra et al., 2002). Reduced amounts of surface-tension lowering large aggregates (LA) of surfactant, resulting from pronounced aggregate conversion to inactive small aggregates (SA), were accompanied by larger amounts of ROS susceptible phospholipid species (Fessler and Summer, 2016). Increased uptake of SA by AMs in combination with SP-C disrupted cholesterol metabolism, which may partly explain the presence of foamy macrophages in the SP-C deficient lung and lung diseases related to SP-C mutated forms (Glasser et al., 2003; Hamvas et al., 2004; Lawson et al., 2004; Stevens et al., 2005; Henderson et al., 2013; Liptzin et al., 2015; Salerno et al., 2016).

The relevance of inflammasome activation and IL-1β signaling in fibrotic parenchyma remodeling has been shown for silica induced- ILDs (Cassel et al., 2008), bleomycin-induced lung fibrosis (Gasse et al., 2007) and various other organ systems (Vilaysane et al., 2010; Wree et al., 2014; Lv et al., 2018). NLRP3 associated IL-1β release mediates NFκβ activation via IL-1R1/myeloid differentiation primary response 88 (MyD88) related signal transduction in fibroblasts. This results in a misbalance between TIMP-1 and matrix-metalloprotease 9 (MMP-9) and 13 (MMP-13) promoting interstitial collagen deposition and fibrosis (So et al., 2007; Ertunc and Hotamisligil, 2016). Furthermore, genetic deletion of NLRP3-inflammasome associated genes in a ventilated-induced fibrosis model resulted in decreased rates of stretch-induced EMT, which was accompanied by ameliorated fibrotic lung remodeling (Lv et al., 2018).

## SP-C, Cholesterol and Lung Mechanics. Evidences From Animal Models and Human Patients

All the molecular and cellular mechanisms explained in the previous sections impact the health and mechanical properties of the lungs. We described in this section the results of deleting the expression or mutating SP-C in animal models (Table 1). In addition, we present here evidences from ILD patients, described to have different mutations in SP-C (Table 2).

**TABLE 1:** SP-C related mouse models.

Mouse model		General results	Lung morphology	BALF	Lung mechanics
**SP-C null mutants**					
Glasser et al., 2001	Generation of SP-C null mutant mice, Swiss black background	Viable, normal growth and reproducibility Reduced stability of small bubbles but normal activity at standard bubble size	Indistinguishable from controls		Reduced hysteresitivity at each end-expiratory pressure
Glasser et al., 2003	SP-C null mutant mice, 129/Sv background	Reduced health and fecundity	From 2 months: enlargement of alveoli, irregular alveolar septation, multifocal cellular infiltrates. From 6 month: type 2 cell hyperplasia, interstitial thickening, peribronchiolar and perivascular monocytic infiltration Intracellular lipid inclusions in macrophages and AE2C, cystoplasmic crystals in macrophages	Increased macrophage number	Increased lung volumes at higher pressures, increased hysteresivity, increased airway resistance and tissue damping
**2nd hit models**					
Lawson et al., 2005	Intratracheal bleomycin application, Swiss black background	Higher mortality and weight loss, more pronounced fibrosis and delayed resolution	Increased number of inflammatory cells, fibrotic foci (collagen, fibroblasts, destroyed septa), enhanced collagen deposition; delayed resolution of fibrosis	Increased neutrophil counts	
Madala et al., 2011	Bleomycin and rapamycin, S129S6 background	Preventive and therapeutic treatment with rapamycin failed to reduce bleomycin induced tissue inflammation and collagen deposition			
Glasser et al., 2008	Instillation of *Pseudomonas aeruginosa*, 129S6 and FVB/N strain	Reduced survival of 2-week-old mice, increased bacterial colony counts in 2-week-old 129S6 but not in FVB/N mice	Increased inflammation, tissue and airway infiltrates (neutrophils and enlarged macrophages with cytoplasmic inclusions)	Increased total cell counts: neutrophils; large foamy macrophages	
Glasser et al., 2009	Respiratory syncytial virus infection, 129S6 and FVB/N	Higher susceptibility to RSV and delayed resolution of induced changes in lung morphology in both strains	More extensive interstitial thickening, air space consolidation, goblet cell hyperplasia.	Increased total cell counts: polymorphonuclear leucocytes, lymphocytes, enlarged foamy mononuclear cells	
Glasser et al., 2013b	RSV infection, expression of SP-C inducible by doxycycline (on 129S6; *55.3/Sftpc-/-*)	SP-C expression reduced RSV-induced tissue inflammation and inflammatory cell counts and increased viral clearance	Diffuse alveolar and interstitial infiltrates in doxycycline untreated mice, reduced inflammation in doxycycline treated mice	Reduced total cell counts and percentage of neutrophil counts in doxycycline -treated mice	
Glasser et al., 2013a	LPS challenge, 129S6 background	More intense airway and airspace inflammation, delayed resolution of tissue inflammation	More intense cellular infiltration, perivascular edema, fragmentation of alveolar septa; residual inflammation 30 days post LPS exposure	Increased total cell counts without LPS challenge (reduced by application of Survanta)	
**Models with incomplete proSP-C processing**					
Conkright et al., 2002	Expression of SP-C_24–57_ HA, FVB/N	Delayed/arrested lung development and lethal neonatal respiratory distress syndrome			
Bridges et al., 2003	Deletion of exon 4	Not viable	Fetal lung tissue: disrupted lung organogenesis, branching morphogenesis, dose-dependent cell cytotoxicity		
Lawson et al., 2011	Conditional expression of L188Q upon doxycycline; intratracheal bleomycin	No spontaneous pulmonary fibrosis; more extensive fibrosis in response to bleomycin	Increased apoptosis, total lung collagen, higher number of myofibroblasts after bleomycin	Cell numbers unaltered in bleomycin treated WT and mutant mice	More reduced static lung compliance in bleomycin treated L188Q mice than challenged controls
Nureki et al., 2018	Conditional mouse mutant, constitutive and inducible I73T expression (by Tamoxifen), C57BL/6J	Increased early mortality, spontaneous acute alveolitis, parenchymal injury, fibrotic remodeling	Constitutive I73T expression: diffuse parenchymal lung remodeling; disrupted embryonic lung architecture Induced expression: acute, diffuse lung injury after tamoxifen, partial recovery but development of fibrotic phenotype	Constitutive expression: age-dependent increases in BALF cellularity induced expression: increased total cell counts, early macrophage accumulation, followed by polymorphonuclear cells and eosinophilia, milder increase in total lymphocytes	Induced expression: restrictive pattern (PV loops), decreased static compliance
Venosa et al., 2019	Conditional mouse mutant, I73T expression induced by Tamoxifen; Local and i.v application of clodronate	Multiphasic and multicellular alveolitis; local clodronate application reduced survival, i.v. clodronate improved survival and reduced eosinophilia		Early reduction of macrophages, followed by accumulation of immature macrophages, neutrophils and eosinophils	
Katzen et al., 2019	Constitutive and conditional C121G mutant inducible by tamoxifen, C57BL/6J	Constitutive expression: lethal postnatal respiratory failure Conditional expression in adult mice: dose-dependent morbidity and mortality, multiphasic polycellular alveolitis with increased BALF cell counts	Constitutive: distorted architecture, enlarged airspaces, interstitial widening, inflammatory infiltrates, proteinaceous fluid conditional expression: acute diffuse lung injury, partial recovery but spontaneous fibrotic lung remodeling	Conditional expression: polycellular alveolitis, increased total cell counts, early macrophage increase, followed by neutrophils and eosinophils, milder increase in lymphocytes	Restrictive pattern: decline in static lung compliance
Jin et al., 2018	Sterile injury model (surfactant protein C-thymidine kinase) induced by ganciclovir in presence (SPC-TK) and absence (SPC-TK/SPC-KO) of SP-C expression	Increased injury and higher mortality in absence than in presence of SP-C expression	Diffuse alveolar damage qualitatively similar but more pronounced in SPC-TK/SPC-KO	Total cell counts unaltered in SPC-TK/SPC-KO and SPC-TK, higher neutrophils and lymphocyte cell counts in SPC-TK/SPC-KO	

**TABLE 2:** Lung mechanics and BALF cells data from patients.

Variant	BALF cells	Lung mechanics	Reference
L188Q		TLC 52%, DLCo 51% (male patient, onset 20 years); FVC 21% (female patient, onset 17 years)	Thomas et al., 2002
I73T	85% M, 12% L, 3% N		Tredano et al., 2004
R167Q	84% M, 11% L, 5% N		
I73T	92% M, 7% N, 1% L, 0% E	FRC: 69% (8 months), 77% (13 years) DLCo: 25% (8 months), 51% (13 years)	Abou Taam et al., 2009
I73T	30% M, 60% N, 10% L, 0% E (Moraxella catarrhalis)	FRC: 138% (36 months), DLCo: 111% (33 months), 128% (36 months), 156% (42 months)	
I73T	82% M, 13% N, 3% L, 2% E	FRC: 120% (26 months), 128% (35 months), 73% (39 months) DLCo: 98% (26 months), 89% (35 months), 164% (39 months)	
I73T	84% M, 5% N, 11% L, 0% E		
I73T	93% M, 1% N, 6% L, 0% E	FRC 112% (26 months), DLCo: 87% (26 months)	
15x I73T, 1x V39A, c.325- 1G > A, c.424delC, c.435G > C (Q145H), L188P, C189Y, L194P	70 ± 5% M, 8 ± 2% L, 18 ± 4% N, total: 379 ± 56 × 10^3^	82% patients with SpO_2_ testing <95%	Thouvenin et al., 2010
I73T	40% M, 57% N, 3% L (mother 32 years)	FVC 62%, TLC 77%, FEV1 83%, RV 108%, DLCo 33%, PaO_2_ room air 11.3 kPa, PaO_2_ after 10 min exercise (35W): 7.3 kPa	Cottin et al., 2011
	74% M, 20% N, 4% L, 2% E (child, 3 months)		
G100S	BAL cell count (100.000 cells/ml): 2.4, 90% M, 7.5% L, 2.5% N, 0% E, CD4/CD8 ratio: 1,7	VC 72.2%, FEV1 84.1% DLCo: 69.3%	Ono et al., 2011
	BAL cell count (100.000 cells/ml): 2, 86% M, 12% L, 1% N, 1% E, CD4/CD8 ratio: 1.6	VC 85%, FEV1 90.3% DLCo: not available	
	BAL cell count (100.000 cells/ml): 1.4, 91% M, 5.8% L, 2.4% N, 0.8% E; CD4/CD8 ratio: 1.5	VC 96.6%, FEV1 85% DLCo: 65.2%	
	BAL cell count(100.000 cells/ml): 1.21,: 54.2% M, 10.1% L, 34.5% N, 1.2% E, CD4/CD8 ratio: 0.25	VC 42.5%, FEV1 92.9% DLCo: 38.5%	
	BAL cell count (100.000 cells/ml): 3.85, 80% M, 17.3% L, 1.1% N, 1.6% E, CD4/CD8 ratio: 0.6 (time diagnosis)	VC 65.3%, FEV1 83.3% DLCo: not available (at time diagnosis)	
Y104H	91% M, 8% L, 1% N	FVC 85%, DLCo 89%, oxygen saturation 97% to 95% (with exercise)	Kuse et al., 2013
I73T		16 years: 90% FVC, 86% TLC, 96% DLCo, 96% VO_2_ max; 37 years: FVC 65%, TLC 91%, DLCo 42%, VO_2_max: 5	Avital et al., 2014
I38F		14 years: FVC 77%, TLC 90%, DLCo 108%, VO_2_ max 78%; 32 years: FVC 94%, TLC 96%, DLCo 82%, VO_2_max 69%, high breathing reserve: 115 l/min, saturation 100% at peak exercise	
I73T		7 years: FVC 59%, TLC 95% DLCo not available, VO_2_max 80%, 28 years: FVC 46%, TLC 48%, DLCo 58%, VO_2_max 79%	
I73T		8 years: FVC 69%, TLC 100%, DLCo 107%, VO_2_ max 83%, 29 years: FVC 102%, TLC 106%, DLCo 95%, VO_2_max 83%	
V39L		16 years: FVC 88%, TLC 95%, DLCo 109%, VO_2_max 93%; 37 years: 94% FVC, 96% TLC, 82% DLCo, 91% VO_2_max	
C121F	Infiltration of granulocytes and alveolar macrophages		van Hoorn et al., 2014
I73T		4 months: 88% oxygen saturation, respiratory rate 85, V_T_ 6.0 ml/kg, V_E_ 507 ml/min/kg, Crs 2.96 ml/cmH_2_O, Crs/kg 0.76/kg, VC 92 ml(52%), TLC 196 ml(74%), FRC 128 ml(110%), RV 104 ml(99%), V_max_FRC 416 ml/s (263%), FEF_75_ 410 ml/s (207%), FEF_85_ 295 ml/s (258%)	Hevroni et al., 2015
I38F		3.3 months: 91% oxygen saturation, respiratory rate 77, V_T_ 6.3 ml/kg, V_E_ 484 ml/min/kg, Crs 2.26 ml/cmH_2_O, Crs/kg 0.59/kg, VC 28 ml(69%), TLC 211 ml(94%), FRC 138 ml(125%), RV 108 ml(109%), V_max_FRC 343 ml/s (245%), FEF_75_ 579 ml/s (334%), FEF_85_ 477 ml/s (476%)	
I73T	Normal cytology and lipid index lipid-laden alveolar macrophages		Salerno et al., 2016
L188E		Normal lung volumes, diffusion capacity 18% of predicted	Chibbar et al., 2004
E66K	Increased cellularity with foamy mononuclear cell		Stevens et al., 2005

*Summary of data from patients suffering fibrosis related to a SP-C mutation. Changes in BALF cells and lung mechanics are summarized for the available data. Crs, respiratory system compliance; PaO_2_, partial pressure of oxygen; RV, residual volume; VC, vital capacity; V_*E*_, minute ventilation; V_*T*_, tidal volume; VO_2_ max, maximal oxygen uptake; FEF_75_, Forced expiratory flow at 75% of forced vital capacity; FEF_85_, Forced expiratory flow at 85% of forced vital capacity. M, macrophage; N, neutrophil; L, lymphocyte; E, eosinophil.*

### SP-C Modifications in Animal Models

Glasser et al. (2001, 2003) reported the generation of SP-C null mutant mice deficient in the expression of *Sftpc* in a Swiss black background. SP-C deficient mice were viable at birth and did grow and reproduce normally with only mild alterations in lung mechanics. In addition, *in vitro* studies of the SP-C deficient derived surfactant proved reduced stability of small bubbles but normal activity at normal bubble size using a captive bubble surfactometer (Glasser et al., 2001, 2003). In contrast, in 129/Sv background, SP-C deficient mice presented with severe morphological changes including enlargement of alveoli, AE2C hyperplasia and interstitial thickening together with a peribronchiolar and perivascular monocyte infiltration in some animals. Although areas of thickened alveolar septa stained positive for alpha-smooth muscle actin and blue in trichrome staining, septa were thinner in regions of airspace enlargement and total lung hydroxyproline remained unaffected (Glasser et al., 2003; Ruwisch et al., 2020). Importantly, lung structure was normal at birth, but emphysema and remodeling developed upon aging suggesting a process of ongoing injury and repair. Investigation of pulmonary mechanics exhibited increased lung volumes at higher pressures as well as increased hysteresivity in SP-C deficient mice. Abnormal intracellular lipid inclusions and crystals were observed in macrophages and AE2C (Glasser et al., 2001, 2003). While phospholipid content was normal in SP-C deficient lungs on the swiss black background, it was 2-fold elevated in 129/Sv background. In addition, lung mechanics showed increased lung volume at high pressure, tissue damping and hysteresivity in 129Sv background. Together, both studies show strain specific influences on the severity of the pulmonary phenotype (Glasser et al., 2001, 2003). Interestingly, as mentioned before, the 129Sv strain demonstrates an increased absorption of dietary cholesterol (Jolley et al., 1999) which might also contribute to the strain specific phenotype in *Sftpc* null mice.

Further investigations included additional hits aggravating the effects of SP-C deficiency (Lawson et al., 2005; Glasser et al., 2008, 2009, 2013a,b). Intratracheal application of bleomycin led to higher mortality, more pronounced weight loss, increased neutrophil counts and enhanced collagen deposition in SP-C deficient mice compared to controls. Increased fibrosis as additionally indicated by morphological parameters went along with a delayed resolution of bleomycin-induced fibrosis (Lawson et al., 2005). Preventive and therapeutic treatment with rapamycin failed to reduce bleomycin induced tissue inflammation and collagen deposition in SP-C deficiency (Madala et al., 2011). Instillation of *Pseudomonas aeruginosa* increased pulmonary injury and bacterial colony counts in SP-C deficient mice compared to control mice. As for unchallenged SP-C deficient mice, sensitivity to *Pseudomonas* challenge seemed to be strain specific since bacterial cell counts were enhanced in 129/Sv6 strain but unaltered in age-matched FVB/N mice. Further, neutrophils and enlarged macrophages with cytoplasmic inclusions were observed upon *Pseudomonas aeruginosa* challenge in absence of SP-C whereby the phagocytic index of macrophages in the SP-C deficient mouse model was reduced (Glasser et al., 2008). Similarly, SP-C deficient mice were more susceptible to respiratory syncytial virus (RSV) infection and resolution of RSV-induced alterations in lung morphology was delayed. Mice exhibited more extensive interstitial thickening, air space consolidation and goblet cell hyperplasia. Polymorphonuclear and macrophage cell counts were increased in BALF obtained from SP-C deficient mice as well as viral titers in lung homogenate (Glasser et al., 2008, 2009). In contrast to challenge with *Pseudomonas aeruginosa*, increased susceptibility of SP-C deficient mice to RSV was not restricted to 129/Sv6 strain but also observed in FVB/N background (Glasser et al., 2008, 2009). However, induction of SP-C expression in compound transgenic mice could reduce RSV-induced tissue inflammation and inflammatory cell counts (Glasser et al., 2013b). LPS-challenged mice develop more intense airway and airspace inflammation in the absence of SP-C. While control mice demonstrate a rapid resolution of LPS-induced cellular and tissue inflammation, inflammation persisted longer in SP-C deficient mice. Cell culture experiments further demonstrated increased LPS-induced cytokine expression of SP-C deficient AE2C compared to those from controls (Glasser et al., 2013b).

The model studied by Glasser et al. (2001, 2008) represents a null mutant mouse model without sufficient *Sftpc* expression leading to the absence of SP-C and proSP-C. Other models include mutations that cause aberrant accumulation of SP-C precursor proteins. Deletion of exon 4 (g.1728 G3A), a mutation detected in human SFTPC, resulted in a truncated form of SP-C (SP-CΔexon4) and incomplete pro-protein processing. Affected mice exhibited disrupted lung organogenesis, branching morphogenesis and expression-depended epithelial cell cytotoxicity (Bridges et al., 2003). Further, transgenic mice that express the mutant L188Q SP-C did not develop pulmonary fibrosis spontaneously, despite induction of ER stress, but developed a more extensive pulmonary fibrosis with reduced compliance and enhanced AEC apoptosis in response to bleomycin and authors suggested alveolar epithelial ER stress to play a crucial role in enhanced disease development (Lawson et al., 2011). Recently, Nureki et al. (2018) developed a conditional mouse mutant in which the expression of I73T, the most common SFTPC mutation found in human patients, is regulated by tamoxifen. In their study, expression of the human mutation induced a spontaneous acute alveolitis, followed by parenchymal injury and fibrotic remodeling in mice (Nureki et al., 2018). Tamoxifen controlled expression of isoleucin to threonine substitution at codon 73 in *Sftpc* (I73T) results in misprocessed proSP-C and leads to increased mortality at day 7 to 14 and acute diffuse parenchymal lung injury with polycellular alveolitis. An early influx of polymorphonuclear cells and macrophages was followed by eosinophilia. Partial recovery from acute injury was followed by aberrant remodeling, collagen deposition and AE2C cell hyperplasia and mechanical abnormalities. Pressure-volume curves displayed a restrictive pattern at 4 to 6 weeks post-tamoxifen and static compliance was reduced at week 4 but rose with resolution of inflammation. However, the fibrotic phenotype was preceded by TGF-β1 (Nureki et al., 2018). A detailed analysis of the same model showed an early reduction of macrophages, followed by an accumulation of immature macrophages and neutrophils before onset of eosinophilia at week 2 in combination with shift from proinflammatory to anti-inflammatory/profibrotic activation state in mRNA BALF cell analysis (Venosa et al., 2019). Total inspiratory capacity and static compliance were reduced in transgenic mice following the induction of the mutant protein whereas tissue damping was elevated, all together suggesting an enhanced tissue stiffness. While local clodronate application diminishing resident macrophages led to reduced survival, intravenous application of clodronate decreased accumulation of immature macrophages, improved survival and reduced eosinophilia (Venosa et al., 2019). Expression of another mutant protein, C121G, resulted in postnatal respiratory failure due to disrupted lung morphogenesis with enlargement of airspaces and interstitial widening in mice constitutively expressing C121G whereas a tamoxifen-mediated expression in adult mice induced acute parenchymal lung injury and a multiphasic polycellular alveolitis with increased BALF cell counts. Similar to the I73T model, an early increase of macrophages and neutrophils was followed by a later influx of eosinophils. Overall, lung mechanics showed a restrictive impairment indicated by a decline in static lung compliance (Katzen et al., 2019).

### SP-C Mutations in Human Patients

Since Nogee et al., (2001) described the first case of a *Sftpc* mutation in an infant girl and her mother diagnosed with ILD (Nogee et al., 2001), several studies have been conducted to provide a link between variants in *SFTPC* and the manifestation of ILDs in adults or children. Since previous groups have already performed systematic reviews describing detected mutations, clinical parameters and radiographic as well as histological findings (Ono et al., 2011; Litao et al., 2017), we decided to focus on the work that reported either BALF cell counting or data on lung mechanics in their study (Table 2). In general, lung mechanics display a restrictive pattern (Thomas et al., 2002; Ono et al., 2011; Kuse et al., 2013; Avital et al., 2014; Hevroni et al., 2015). In particular, vital capacity, forced vital capacity were markedly reduced in some patients (Ono et al., 2011; Kuse et al., 2013; Avital et al., 2014; Hevroni et al., 2015) whereas mild reductions in forced expiratory pressure in 1 s (FEV1) (Cottin et al., 2011; Ono et al., 2011) were attributable to a generally reduced lung volume and not indicative of an obstruction. However, the reported residual volumes were high to normal (Cottin et al., 2011; Hevroni et al., 2015). Changes in functional residual capacity (FRC) were unspecific. While some patients demonstrated a reduced FRC, other studies reported elevated or normal values. Gas exchange as indicated by diffusing capacity for carbon monoxide exhibited distinct impairment in most (Thomas et al., 2002; Abou Taam et al., 2009; Cottin et al., 2011; Ono et al., 2011; Avital et al., 2014) resulting in a generally reduced oxygen (Thouvenin et al., 2010; Hevroni et al., 2015). However, Abou Taam and coworkers reported normal and increased diffusion capacity for carbon monoxide (D_LCO_) in two patients (Abou Taam et al., 2009) and oxygenation was sufficient in another patient reported by Avital et al. (2014). Hence, heterogeneity of the disease is also reflected in lung function. While the majority of studies linked SFTPC mutations to the familial form of pulmonary fibrosis, genetic mutations in SFTPC were also detected in a subset of patients suffering from sporadic IPF (Lawson et al., 2004). Interestingly, sporadic patients also demonstrate with mixed pattern as reported by Cottin et al. (2011). Indeed, they reported a case of combined pulmonary fibrosis and emphysema in a patient with the most common I73T mutation.

As explained before, pulmonary fibrosis and especially IPF was linked to an AE2C dysfunction and increased ER stress response due to aberrant protein accumulation. Hyperplastic AE2C in histological examination are a common finding (Nogee et al., 2001; Chibbar et al., 2004; Hamvas et al., 2004; Cameron et al., 2005; Stevens et al., 2005; Soraisham et al., 2006; Mechri et al., 2010; Thouvenin et al., 2010; Citti et al., 2013; Litao et al., 2017; Park et al., 2018) and enhanced ER stress is a widely acknowledged pathomechanism in IPF (Korfei et al., 2008). However, reduced transcription of SFTPC due to promotor variant were associated with neonatal respiratory distress syndrome in late preterm infants (Wambach et al., 2010) and SP-C was absent in BALF samples of a family with chronic ILDs, together with reduced pro-SP-C staining, though no mutation was identified (Amin et al., 2001). While the majority of mutations leads to the onset of ILDs already in childhood (Chibbar et al., 2004; Hamvas et al., 2004; Tredano et al., 2004; Rosen and Waltz, 2005; Stevens et al., 2005; Bullard and Nogee, 2007; Guillot et al., 2009; Mechri et al., 2010; Cottin et al., 2011; Citti et al., 2013; Henderson et al., 2013; Hepping et al., 2013; Turcu et al., 2013; Akimoto et al., 2014; Avital et al., 2014; van Hoorn et al., 2014; Kroner et al., 2015; Liptzin et al., 2015; Peca et al., 2015; Griese et al., 2016; Liu et al., 2016; Hayasaka et al., 2018) a subset of variants results in a manifestation in later child- or adulthood (Lawson et al., 2004; Setoguchi et al., 2006; Markart et al., 2007; van Moorsel et al., 2010; Cottin et al., 2011; Ono et al., 2011; Kuse et al., 2013) suggesting a chronic process.

Besides to AE2C hyperplasia, cholesterol clefts are frequently detected on lung biopsy in context of an SFTPC mutations (Hamvas et al., 2004; Abou Taam et al., 2009; Mechri et al., 2010; Cottin et al., 2011; Litao et al., 2017) or on lung specimen from ILD patients lacking SP-C (Amin et al., 2001). Macrophages were often described as foamy (Lawson et al., 2004; Stevens et al., 2005; Henderson et al., 2013) or lipid-laden (Liptzin et al., 2015; Salerno et al., 2016). When analyzing BALF cells, the majority of cells usually consists of macrophages (Abou Taam et al., 2009; Thouvenin et al., 2010; Ono et al., 2011). However, some samples showed a marked increase in neutrophil (Thouvenin et al., 2010; Cottin et al., 2011) or lymphocyte (Ono et al., 2011) cell counting in the absence of an infection. Whether immune cell infiltration occurs in reaction to abnormal histological findings or rather causes alterations remains unclear in view of single time point studies in human patients. In addition, a detailed characterization of macrophages was not provided by the majority of studies, but different activation could have distinct effects on fibrogenesis.

## Lung Fibrosis and Cholesterol

Recently, we have described the accumulation of cholesterol crystals in AMs of SP-C deficient animals (Ruwisch et al., 2020). We also showed similar crystals accumulated in the lung of an IPF patient. Looking through the literature this feature is commonly described (Glasser et al., 2003; Hoenerhoff et al., 2006; Mora et al., 2006) but no further investigated. Therefore, the role of cholesterol or accumulation of cholesterol in the form of crystals in cells is yet not well understood and often overlooked. As explained before, cholesterol is also involved in many processes apart from regulating membrane biophysical properties. Not many experimental data are available in the context of the influence of cholesterol in lung diseases. However, in the clinic settings, it is not surprising that certain patients are suffering from comorbidities that should be treated such as hypercholesterolemia. In fact, it is known that IPF increases the risk of heart diseases. Thus, it is common for IPF patients to receive medications aimed at reducing cardiovascular risk, including statins, which lower cholesterol levels (Kreuter et al., 2018). Data in this regard is still quite controversial. While, 3-hydroxy-3-methylglutaryl-coenzyme A reductase inhibitors (statins) aggravated pulmonary fibrosis in mice by increased production of mitochondrial ROS and NLRP3 inflammasome activation in macrophages. Statins, as potent inhibitors of the endogenous cholesterol synthesis have been associated with a lower decline in FVC in a cohort of untreated IPF patients (Kreuter et al., 2018). In line with this, diet induced systemic hyperlipidemia resulted in altered surfactant phospholipid composition leading to increased alveolar collapsibility, providing evidence that pulmonary lipid homeostasis is strongly related to the blood circulation (Baritussio et al., 1980). As approximately 83% of surfactant cholesterol has been stated to be derived from blood plasma (Turley et al., 1981), statins could contribute in preventing deleterious high levels of cholesterol in surfactant films, providing surfactant film stability and avoiding alveolar collapse. In addition, evaluation of lung function showed that patients receiving statin therapy and Ofev had less decline in pulmonary capacity, compared with a placebo (Kreuter et al., 2018). The yardstick that researchers used was patients’ annual rate of decline in forced vital capacity (FVC). The results suggested that statins do not diminish Ofev’s effectiveness (Kreuter et al., 2018).

Statins may also have an anti-inflammatory effect in the lung (Liao and Laufs, 2004; Jain and Ridker, 2005), thus a multifactorial mechanism may play an important role in their use. In conclusion, more experimental evidence is needed to support the use of cholesterol lowering drugs for the treatment of lung diseases such as fibrosis. In addition, the role of cholesterol and SP-C has to be further described and understood in order to develop new therapies. However, there is already promising data in this regard, opening a new pathway to target.

## Conclusion

Summing up there are various potential either direct or indirect mechanisms how SP-C is able to interfere and modulate fibrogenesis in the lung. While SP-C deficiency mediated surfactant dysfunction and impaired lung mechanics are likely contributors in the generation of local mechanical stresses and strains, its biophysical interplay with cholesterol on the one hand, and its modulation of TLR-4 signaling on the other hand, highlights SP-C as a key element in a complex profibrotic network. Thereby SP-C deficiency comes into play not only in animal models, but also in patients with familial and non-familial forms of lung fibrosis such as IPF. Further research is needed in order to determine the potential of cholesterol lowering drugs as treatment or combined with anti-fibrotic drugs to find a better therapy for IPF patients.

## Author Contributions

KS, JR, NR, and EL-R contributed to the review, writing, and revising of the manuscript.

## Conflict of Interest

NR was employed by the company AlveoliX AG.

The remaining authors declare that the research was conducted in the absence of any commercial or financial relationships that could be construed as a potential conflict of interest.

## Abbreviations

α-SMA: alpha smooth muscle actin
aaAMs: alternatively activated alveolar macrophages
ABCA1: ATP binding cassette transporter A1
ABCG1: ATP binding cassette transporter G1
ADA: adenosine-deaminase
ADP: adenosine diphosphate
AEC: alveolar epithelial cell
AE1C: alveolar epithelial type 1 cell
AE2C: alveolar epithelial type 2 cell
ALI: acute lung injury
AM: alveolar macrophages
AMP: adenosine monophosphate
ApoA1: apolipoprotein A1
ApoE: apolipoprotein E
ARDS: acute respiratory distress syndrome
Arg-1: arginase 1
ASC: apoptosis speck-like protein
ATP: adenosine triphosphate
BALF: bronchoalveolar lavage fluid
Ca^2+^: calcium
caAMs: classically activated alveolar macrophages
CARD: caspase recruitment domain
chILD: Children Interstitial Lung Disease
DAMPs: damage associated molecular patterns
D_LCO_: diffusion capacity for carbon monoxide
DPPC: dipalmitoylphosphatidylcholine
EC: extracellular
ECM: extracellular matrix
EMT: epithelial to mesenchymal transition
ER: endoplasmic reticulum
FEV1: forced expiratory pressure in 1 second
FRC: forced residual capacity
GOE: great oxygen event
HCl: chloride acid
HPS: Hermansky–Pudlak syndrome
HRCT: high resolution computed tomography
ILD: interstitial lung disease
IPF: idiopathic pulmonary fibrosis
LA: large aggregate
LAP-1: latency associated peptide 1
LDL: low-density lipoprotein
LPS: lipopolysaccharide
LTP-1: latency TGF- β 1 binding protein-1
mRNA: messenger ribonucleic acid
MMP-9: matrix-metalloprotease 9
MMP-13: matrix-metalloprotease 13
MyD88: myeloid differentiation primary response 88
NF κβ: necrosis factor-kappa-beta
NLRP3 NOD-: LRR- and pyrin domain-containing protein 3
NOS-2: inducible nitric oxide synthase
NRDS: neonatal respiratory distress syndrome
PAP: pulmonary alveolar proteinosis
PC: phosphatydilcholine
PEEP: positive end-expiratory pressure
P2XR7: P2X purinoreceptor 7
P2 × 4R: P2X purinoreceptor 4
P2Y2R: P2Y purinoreceptor 2
Relm- α: resistin-like alpha
ROS: reactive oxygen species
RSV: respiratory syncytial virus
SA: small aggregate
SHH: sonic hedgehog
SP-B: surfactant protein B
SP-C: surfactant protein C
SFTPC: surfactant protein C gene
TGF-b1: transforming growth factor beta 1
TIMP1: tissue-metalloprotease inhibitor 1
TLC: total lung capacity
TLR-4: Toll-like receptor 4
TNF- α: tumor necrosis factor alpha
UIP: unusual interstitial pneumonia
VILI: ventilation induced lung injury
YM1: chitinase-like protein 1.
