# Corrigendum: Alveolar Dynamics and Beyond – the Importance of Surfactant Protein C and Cholesterol in Lung Homeostasis and Fibrosis

**DOI:** 10.3389/fphys.2020.00943

**Published:** 2020-09-15

**Authors:** Kirsten Sehlmeyer, Jannik Ruwisch, Nuria Roldan, Elena Lopez-Rodriguez

**Affiliations:** ^1^Institute of Functional and Applied Anatomy, Hannover Medical School, Hanover, Germany; ^2^Biomedical Research in Endstage and Obstructive Lung Disease Hannover, Member of the German Centre for Lung Research, Hanover, Germany; ^3^Alveolix AG and ARTORG Center, University of Bern, Bern, Switzerland; ^4^Institute of Functional Anatomy, Charité – Universitätsmedizin Berlin, Berlin, Germany

**Keywords:** surfactant protein C, pulmonary fibrosis, alveolar dynamics, lipid metabolism, alveolar macrophages, cholesterol, metaflammation

In the original article, there was a mistake in Table 1 and Table 2 as published. **References on tables are misplaced**.

In Table 1, the cited reference Glasser et al., 2008 should be Glasser et al., 2001. The cited reference Glasser and Senft, 2009 should be Glasser et al., 2003. The cited reference Glasser et al., 2013b should be Lawson et al., 2005. The cited reference Glasser et al., 2013a should be Madala et al., 2011. The cited references Bridges et al., 2003 should be Glasser et al., 2008. The cited reference Lawson et al., 2011 should be Glasser et al., 2009. The cited reference Nureki et al., 2018 should be Glasser et al., 2013b. The cited reference Venosa et al., 2019 should be Glasser et al., 2013a. The cited reference Katzen et al., 2019 should be Bridges et al., 2003. The cited reference Jin et al., 2018 should be Lawson et al., 2011. The cited reference Conkright et al., 2002 in the third row (section Models with incomplete proSP-C processing) should be Nureki et al., 2018. The cited reference Conkright et al., 2002 in the fourth row should be Venosa et al., 2019. The cited reference Conkright et al., 2002 in the fifth row should be Katzen et al., 2019. The cited reference Conkright et al., 2002 in the sixth row should be Jin et al., 2018. And the cited reference Thouvenin et al., 2010 should be Conkright et al., 2002.

In Table 2, the cited reference Cottin et al., 2011 should be Thomas et al., 2002. The cited reference Ono et al., 2011 should be Tredano et al., 2004. The cited reference Kuse et al., should be Abou Taam et al., 2009. The cited reference Avital et al., 2014 should be Thouvenin et al., 2010. The cited reference van Hoorn et al., 2014 should be Cottin et al., 2011. The cited reference Hevroni et al., 2015 should be Ono et al., 2011. The cited reference Salerno et al., 2016 should be Kuse et al., 2013. The cited reference Chibbar et al., 2004 should be Avital et al., 2014. The cited reference Stevens et al., 2005 should be van Hoorn et al., 2014. The cited reference Glasser et al., 2001 should be Hevroni et al., 2015. The cited reference Glasser et al., 2003 should be Salerno et al., 2016. The cited reference Lawson et al., 2005 should be Chibbar et al., 2004. And the last cited reference Lawson et al., 2005 should be Stevens et al., 2005. The corrected Table 1 and Table 2 appears below.

**Table 1:** SP-C related mouse models.

**Mouse model**		**General results**	**Lung morphology**	**BALF**	**Lung mechanics**
**SP-C null mutants**					
Glasser et al., 2001	Generation of SP-C null mutant mice, Swiss black background	Viable, normal growth and reproducibility.Reduced stability of small bubbles but normal activity at standard bubble size	Indistinguishable from controls		Reduced hysteresitivity at each end-expiratory pressure
Glasser et al., 2003	SP-C null mutant mice, 129/Sv background	Reduced health and fecundity	From 2 month: enlargement of alveoli, irregular alveolar septation, multifocal cellular infiltrates. From 6 month: type 2 cell hyperplasia, interstitial thickening, peribronchiolar and perivascular monocytic infiltration. Intracellular lipid inclusions in macrophages and AE2C, cytoplasmic crystals in macrophages	Increased macrophage number	Increased lung volumes at higher pressures, increased hysteresivity, increased airway resistance and tissue damping
**2nd hit models**					
Lawson et al., 2005	Intratracheal bleomycin application, Swiss black background	Higher mortality and weight loss, more pronounced fibrosis and delayed resolution	Increased number of inflammatory cells, fibrotic foci (collagen, fibroblasts, destroyed septa), enhanced collagen deposition; delayed resolution of fibrosis	Increased neutrophil counts	
Madala et al., 2011	Bleomycin and rapamycin, S129S6 background	Preventive and therapeutic treatment with rapamycin failed to reduce bleomycin induced tissue inflammation and collagen deposition			
Glasser et al., 2008	Instillation of *Pseudomonas aeruginosa*, 129S6 and FVB/N strain	Reduced survival of 2-week-old mice, increased bacterial colony counts in 2-week-old 129S6 but not in FVB/N mice	Increased inflammation, tissue and airway infiltrates (neutrophils and enlarged macrophages with cytoplasmic inclusions)	Increased total cell counts: neutrophils; large foamy macrophages	
Glasser et al., 2009	Respiratory syncytial virus infection, 129S6 and FVB/N	Higher susceptibility to RSV and delayed resolution of induced changes in lung morphology in both strains	More extensive interstitial thickening, air space consolidation, goblet cell hyperplasia	Increased total cell counts: polymorphonuclear leucocytes, lymphocytes, enlarged foamy mononuclear cells	
Glasser et al., 2013b	RSV infection, expression of SP-C inducible by doxycycline (on 129S6; *55.3/Sftpc-/-*)	SP-C expression reduced RSV-induced tissue inflammation and inflammatory cell counts and increased viral clearance	Diffuse alveolar and interstitial infiltrates in doxycycline untreated mice, reduced inflammation in doxycycline treated mice	Reduced total cell counts and percentage of neutrophil counts in doxycycline -treated mice	
Glasser et al., 2013a	LPS challenge, 129S6 background	More intense airway and airspace inflammation, delayed resolution of tissue inflammation	More intense cellular infiltration, perivascular edema, fragmentation of alveolar septae; residual inflammation 30 days post LPS exposure	Increased total cell counts without LPS challenge (reduced by application of Survanta)	
**Models with incomplete proSP-C processing**					
Conkright et al., 2002	Expression of SP-C_24−57_ HA, FVB/N	Delayed/arrested lung development and lethal neonatal respiratory distress syndrome			
Bridges et al., 2003	Deletion of exon 4	Not viable	Fetal lung tissue: disrupted lung organogenesis, branching morphogenesis, dose-dependent cell cytotoxicity		
Lawson et al., 2011	Conditional expression of L188Q upon doxycycline; intratracheal bleomycin	No spontaneous pulmonary fibrosis; more extensive fibrosis in response to bleomycin	Increased apoptosis, total lung collagen, higher number of myofibroblasts after bleomycin	Cell numbers unaltered in bleomycin treated WT and mutant mice	More reduced static lung compliance in bleomycin treated L188Q mice than challenged controls
Nureki et al., 2018	Conditional mouse mutant, constitutive and inducible I73T expression (by Tamoxifen), C57BL/6J	Increased early mortality, spontaneous acute alveolitis, parenchymal injury, fibrotic remodeling	Constitutive I73T expression: diffuse parenchymal lung remodeling; disrupted embryonic lung architecture Induced expression: acute, diffuse lung injury after tamoxifen, partial recovery but development of fibrotic phenotype	Constitutive expression: age-dependent increases in BALF cellularity Induced expression: increased total cell counts, early macrophage accumulation, followed by polymorphonuclear cells and eosinophilia, milder increase in total lymphocytes	Induced expression: restrictive pattern (PV loops), decreased static compliance
Venosa et al., 2019	Conditional mouse mutant, I73T expression induced by Tamoxifen; Local and i.v application of clodronate	Multiphasic and multicellular alveolitis; local clodronate application reduced survival, i.v. clodronate improved survival and reduced eosinophilia		Early reduction of macrophages, followed by accumulation of immature macrophages, neutrophils and eosinophils	
Katzen et al., 2019	Constitutive and conditional C121G mutant inducible by tamoxifen, C57BL/6J	Constitutive expression: lethal postnatal respiratory failure Conditional expression in adult mice: dose-dependent morbidity and mortality, multiphasic polycellular alveolitis with increased BALF cell counts	Constitutive: distorted architecture, enlarged airspaces, interstitial widening, inflammatory infiltrates, proteinaceous fluid Conditional expression: acute diffuse lung injury, partial recovery but spontaneous fibrotic lung remodeling	Conditional expression: polycellular alveolitis, increased total cell counts, early macrophage increase, followed by neutrophils and eosinophils, milder increase in lymphocytes	Restrictive pattern: decline in static lung compliance
Jin et al., 2018	Sterile injury model (surfactant protein C-thymidine kinase) induced by ganciclovir in presence (SPC-TK) and absence (SPC-TK/SPC-KO) of SP-C expression	Increased injury and higher mortality in absence than in presence of SP-C expression	Diffuse alveolar damage qualitatively similar but more pronounced in SPC-TK/SPC-KO	Total cell counts unaltered in SPC-TK/SPC-KO and SPC-TK, higher neutrophils and lymphocyte cell counts in SPC-TK/SPC-KO	

**Table 2:** Lung mechanics and BALF cells data from patients.

**Variant**	**BALF cells**	**Lung mechanics**	**Reference**
L188Q		TLC 52%, DLCo 51% (male patient, onset 20 years); FVC 21% (female patient, onset 17 years)	Thomas et al., 2002
I73T	85% M, 12% L, 3% N		Tredano et al., 2004
R167Q	84% M, 11% L, 5% N		
I73T	92% M, 7% N, 1% L, 0% E	FRC: 69% (8 months), 77% (13 years)DLCo: 25% (8 months), 51% (13 years	Abou Taam et al., 2009
I73T	30% M, 60% N, 10% L, 0% E (Moraxella catarrhalis)	FRC: 138% (36 months), DLCo: 111% (33 months), 128% (36 months), 156% (42 months)	
I73T	82% M, 13% N, 3% L, 2% E	FRC: 120% (26 months), 128% (35 months), 73% (39 months) DLCo: 98% (26 months), 89% (35 months), 164% (39 months)	
I73T	84% M, 5% N, 11% L, 0% E		
I73T	93% M, 1% N, 6% L, 0% E	FRC 112% (26 months), DLCo: 87% (26 months)	
15x I73T, 1x V39A, c.325- 1G>A, c.424delC, c.435G>C (Q145H), L188P, C189Y, L194P	70 ± 5% M, 8 ± 2% L, 18 ± 4% N, total: 379 ± 56 x 10^3^	82% patients with SpO_2_ testing <95%	Thouvenin et al., 2010
I73T	40% M, 57% N, 3% L (mother 32 years)	FVC 62%, TLC 77%, FEV1 83%, RV 108%, DLCo 33%, PaO_2_ room air 11.3 kPa, PaO_2_ after 10 min exercise (35W): 7.3 kPa	Cottin et al., 2011
	74% M, 20% N, 4% L, 2% E (child, 3 months)		
G100S	BAL cell count (100.000 cells/ml): 2.4, 90% M, 7.5% L, 2.5% N, 0% E; CD4/CD8 ratio: 1,7	VC 72.2%, FEV1 84.1% DLCo: 69.3%	Ono et al., 2011
	BAL cell count (100.000 cells/ml): 2, 86% M, 12% L, 1% N, 1% E, CD4/CD8 ratio: 1.6	VC 85%, FEV1 90.3% DLCo: not available	
	BAL cell count (100.000 cells/ml):1.4, 91% M, 5.8% L, 2.4%N, 0.8%E, CD4/CD8 ratio: 1.5	VC 96.6%, FEV1 85% DLCo: 65.2%	
	BAL cell count (100.000 cells/ml): 1.21, 54.2% M, 10.1%L, 34.5% N, 1.2% E, CD4/CD8 ratio: 0.25	VC 42.5%, FEV1 92.9% DLCo: 38.5%	
	BAL cell count (100.000 cells/ml): 3.85, 80% M, 17.3% L, 1.1% N, 1.6% E, CD4/CD8 ratio: 0.6 (time diagnosis)	VC 65.3%, FEV1 83.3% DLCo: not available (at time diagnosis)	
Y104H	91% M, 8% L, 1% N	FVC 85%, DLCo 89%, oxygen saturation 97% to 95% (with exercise)	Kuse et al., 2013
I73T		16 years: 90% FVC, 86% TLC, 96% DLCo, 96% VO_2_ max; 37 years: FVC 65%, TLC 91%, DLCo 42%, VO_2_max: 5	Avital et al., 2014
I38F		14 years: FVC 77%, TLC 90%, DLCo 108%, VO_2_max 78%; 32 years: FVC 94%, TLC 96%, DLCo 82%, VO_2_max 69%, high breathing reserve: 115 l/min, saturation 100% at peak exercise	
I73T		7 years: FVC 59%, TLC 95% DLCo not available, VO_2_max 80%, 28 years: FVC 46%, TLC 48%, DLCo 58%, VO_2_max 79%	
I73T		8 years: FVC 69%, TLC 100%, DLCo 107%, VO_2_max 83%, 29 years: FVC 102%, TLC 106%, DLCo 95%, VO_2_max 83%	
V39L		16 years: FVC 88%, TLC 95%, DLCo 109%, VO_2_max 93%; 37 years: 94% FVC, 96% TLC, 82% DLCo, 91% VO_2_max	
C121F	Infiltration of granulocytes and alveolar macrophages		van Hoorn et al., 2014
I73T		4 months:88% oxygen saturation, respiratory rate 85, V_T_ 6.0 ml/kg, V_E_ 507 ml/min/kg, Crs 2.96 ml/cmH_2_O, Crs/kg 0.76/kg, VC 92 ml(52%), TLC 196 ml(74%), FRC 128 ml(110%), RV 104 ml(99%), V_max_FRC 416 ml/s (263%), FEF_75_ 410 ml/s (207%), FEF_85_ 295 ml/s(258%)	Hevroni et al., 2015
I38F		3.3 months: 91% oxygen saturation, respiratory rate 77, V_T_ 6.3 ml/kg, V_E_ 484 ml/min/kg, Crs 2.26 ml/cmH_2_O, Crs/kg 0.59/kg, VC 28 ml(69%), TLC 211 ml(94%), FRC 138 ml(125%), RV 108 ml(109%), V_max_FRC 343 ml/s (245%), FEF_75_ 579 ml/s (334%), FEF_85_ 477 ml/s (476%)	
I73T	Normal cytology and lipid index lipid-laden alveolar macrophages		Salerno et al., 2016
L188E		Normal lung volumes, diffusion capacity 18% of predicted	Chibbar et al., 2004
E66K	Increased cellularity with foamy mononuclear cell		Stevens et al., 2005

*Summary of data from patients suffering fibrosis related to a SP-C mutation. Changes in BALF cells and lung mechanics are summarized for the available data. Crs, respiratory system compliance; PaO2, partial pressure of oxygen; RV, residual volume; VC, vital capacity; VE, minute ventilation; VT, tidal volume; VO2 max, maximal oxygen uptake; FEF75, Forced expiratory flow at 75% of forced vital capacity; FEF85, Forced expiratory flow at 85% of forced vital capacity. M, macrophage; N, neutrophil; L, lymphocyte; E, eosinophil*.

In the original article some references are misplaced.

In section “**Inflammatory Response Under Impaired Lung Mechanics – Where Sterols Come into Play**,” **paragraph** 5, the cited reference Ertunc and Hotamisligil, 2016 was incorrectly placed. It should be Jin et al., 2018. In paragraph 6, the cited reference Veldhuizen et al., 1996, 1997; Vazquez De Anda et al., 2000; Maitra et al., 2002 should be Ertunc and Hotamisligil, 2016. The cited reference Fessler and Summer, 2016 should be Veldhuizen et al., 1996, 1997; Vazquez De Anda et al., 2000; Maitra et al., 2002. The cited reference Glasser et al., 2003; Hamvas et al., 2004; Lawson et al., 2004; Stevens et al., 2005; Henderson et al., 2013; Liptzin et al., 2015; Salerno et al., 2016 should be Fessler and Summer, 2016. The cited reference Cassel et al., 2008 should be Glasser et al., 2003; Hamvas et al., 2004; Lawson et al., 2004; Stevens et al., 2005; Henderson et al., 2013; Liptzin et al., 2015; Salerno et al., 2016. In **paragraph 7**, the cited reference Vilaysane et al., 2010; Wree et al., 2014; Lv et al., 2018 should be Cassel et al., 2008. The cited reference Zhou et al., 2013 should be Gasse et al., 2007. The cited reference So et al., 2007; Ertunc and Hotamisligil, 2016 should be Vilaysane et al., 2010; Wree et al., 2014; Lv et al., 2018. The cited reference; Gasse et al., 2007; Robert et al., 2016 should be So et al., 2007; Ertunc and Hotamisligil, 2016.

In section “**SP-C Modifications in Animal Models**,” **paragraph 1**, the cited reference Lawson et al., 2005; Glasser et al., 2008, 2013a,b; Glasser and Senft, 2009; Ruwisch et al., 2020 should be Glasser et al., 2003; Ruwisch et al., 2020. The cited reference Jolley et al., 1999 should be Glasser et al., 2001; Glasser et al., 2003. The cited reference Lawson et al., 2005; Glasser et al., 2008, 2013a,b; Glasser and Senft, 2009 should be Jolley et al., 1999. In **paragraph 2**, the cited reference Lawson et al., 2005 should be Lawson et al., 2005; Glasser et al., 2008; Glasser et al., 2009; Glasser et al., 2013a,b. The cited reference Madala et al., 2011 should be Lawson et al., 2005. The cited reference Glasser et al., 2008 should be Madala et al., 2011. The cited reference Glasser et al., 2009 should be Glasser et al., 2008. The cited reference Glasser and Senft, 2009 should be Glasser et al., 2009. The cited reference Glasser et al., 2013b should be Glasser et al., 2008; Glasser et al., 2009. The cited reference Glasser et al., 2008 should be Glasser et al., 2013b. In **paragraph 3**, the cited reference Glasser et al. (2001), Bridges et al. (2003), should be Glasser et al., 2001, 2008. The cited reference Lawson et al., 2011, should be Bridges et al., 2003; in **line 17**, first Nureki et al., 2018 should be Lawson et al., 2011; in **line 35**, Venosa et al., 2019 should be Nureki et al., 2018. The cited reference Katzen et al., 2019 should be Venosa et al., 2019. The cited reference Nogee et al., 2001 should be Katzen et al., 2019.

In section “**SP-C Mutations in Human Patients**,” **paragraph 1**, the cited reference Ono et al. (2011), Litao et al. (2017) should be Nogee et al., 2001; in **line 3** Thomas et al., 2002; Ono et al., 2011; Kuse et al., 2013; Avital et al., 2014; Hevroni et al., 2015 should be Nogee et al., 2001; in **line 9** Thomas et al., 2002; Ono et al., 2011; Kuse et al., 2013; Avital et al., 2014; Hevroni et al., 2015 should be Ono et al., 2011; Litao et al., 2017. The cited reference Cottin et al., 2011; Ono et al., 2011 should be Ono et al., 2011; Kuse et al., 2013; Avital et al., 2014; Hevroni et al., 2015. The cited reference Cottin et al., 2011, Hevroni et al., 2015 should be Cottin et al., 2011; Ono et al., 2011. The cited reference Thomas et al., 2002; Abou Taam et al., 2009; Cottin et al., 2011; Ono et al., 2011; Avital et al., 2014 should be Cottin et al., 2011; Hevroni et al., 2015. The cited reference Thouvenin et al., 2010; Hevroni et al., 2015 should be Thomas et al., 2002; Abou Taam et al., 2009; Cottin et al., 2011; Ono et al., 2011; Avital et al., 2014, and Abou Taam et al., 2009 should be Thouvenin et al., 2010; Hevroni et al., 2015. The cited reference Avital et al., 2014 should be Abou Taam et al., 2009. The cited reference Lawson et al. (2004) should be Avital et al., 2014. The cited reference Cottin et al., 2011 should be Lawson et al., 2004 and Nogee et al. (2001), Chibbar et al. (2004), Hamvas et al. (2004), Lawson et al. (2004), Cameron et al. (2005), Stevens et al. (2005), Soraisham et al. (2006), Mechri et al. (2010), Thouvenin et al. (2010), Citti et al. (2013), Park et al. (2018) should be Cottin et al., 2011.

In **paragraph 2**, the cited reference Korfei et al., 2008 should be Nogee et al., 2001; Chibbar et al., 2004; Hamvas et al., 2004; Cameron et al., 2005; Stevens et al., 2005; Soraisham et al., 2006; Mechri et al., 2010; Thouvenin et al., 2010; Citti et al., 2013; Litao et al., 2017; Park et al., 2018. The cited reference Wambach et al., 2010 should be Korfei et al., 2008. The cited reference Amin et al., 2001 should be Wambach et al., 2010. The cited reference Chibbar et al., 2004; Hamvas et al., 2004; Tredano et al., 2004; Rosen and Waltz, 2005; Stevens et al., 2005; Bullard and Nogee, 2007; Guillot et al., 2009; Mechri et al., 2010; Cottin et al., 2011; Citti et al., 2013; Henderson et al., 2013; Hepping et al., 2013; Turcu et al., 2013; Akimoto et al., 2014; Avital et al., 2014; van Hoorn et al., 2014; Kroner et al., 2015; Liptzin et al., 2015; Peca et al., 2015; Griese et al., 2016; Liu et al., 2016; Hayasaka et al., 2018 should be Amin et al., 2001. The cited reference Lawson et al., 2004; Setoguchi et al., 2006; Markart et al., 2007; van Moorsel et al., 2010; Cottin et al., 2011; Ono et al., 2011; Kuse et al., 2013 should be Chibbar et al., 2004; Hamvas et al., 2004; Tredano et al., 2004; Rosen and Waltz, 2005; Stevens et al., 2005; Bullard and Nogee, 2007; Guillot et al., 2009; Mechri et al., 2010; Cottin et al., 2011; Citti et al., 2013; Henderson et al., 2013; Hepping et al., 2013; Turcu et al.,2013; Akimoto et al., 2014; Avital et al., 2014; van Hoorn et al., 2014; Kroner et al., 2015; Liptzin et al., 2015; Peca et al., 2015; Griese et al., 2016; Liu et al., 2016; Hayasaka et al., 2018 and Hamvas et al., 2004; Abou Taam et al., 2009; Mechri et al., 2010; Cottin et al., 2011; Litao et al., 2017 should be Lawson et al., 2004; Setoguchiet al., 2006; Markart et al., 2007; van Moorsel et al., 2010; Cottin et al., 2011; Ono et al., 2011; Kuse et al., 2013. In **paragraph 3**, the cited reference Amin et al., 2001 should be Hamvas et al., 2004; Abou Taam et al., 2009; Mechri et al., 2010; Cottin et al., 2011; Litao et al., 2017. The cited reference Lawson et al., 2004; Stevens et al., 2005; Hamvas, 2010; Henderson et al., 2013 should be Amin et al., 2001. The cited reference Liptzin et al., 2015; Salerno et al., 2016 should be Lawson et al., 2004; Stevens et al., 2005; Henderson et al., 2013. The cited reference Tredano et al., 2004; Abou Taam et al., 2009; Thouvenin et al., 2010; Ono et al., 2011 should be Liptzin et al., 2015; Salerno et al., 2016. The cited reference Thouvenin et al., 2010; Cottin et al., 2011 should be Abou Taam et al., 2009; Thouvenin et al., 2010; Ono et al., 2011. The cited reference Tredano et al., 2004; Ono et al., 2011 should be Thouvenin et al., 2010; Cottin et al., 2011 and Ruwisch et al., 2020 should be Ono et al., 2011.

In section “**Lung Fibrosis and Cholesterol**” the cited reference Xu et al., 2012 should be Kreuter et al., 2018. The cited reference Baritussio et al., 1980 should be Kreuter et al., 2018. The cited reference Turley et al., 1981 should be Baritussio et al., 1980. The cited reference Milos et al., 2016 should be Turley et al., 1981. The cited reference Liao and Laufs, 2004; Jain and Ridker, 2005 should be Kreuter et al., 2018 and Thomas et al., 2002 should be Liao and Laufs, 2004; Jain and Ridker, 2005.

The authors apologize for this error and state that this does not change the scientific conclusions of the article in any way. The original article has been updated.

